# Multi-omics quantitative data of tomato fruit unveils regulation modes of least variable metabolites

**DOI:** 10.1186/s12870-023-04370-0

**Published:** 2023-07-22

**Authors:** Annick Moing, Thierry Berton, Léa Roch, Salimata Diarrassouba, Stéphane Bernillon, Stéphanie Arrivault, Catherine Deborde, Mickaël Maucourt, Cécile Cabasson, Camille Bénard, Sylvain Prigent, Daniel Jacob, Yves Gibon, Martine Lemaire-Chamley

**Affiliations:** 1 INRAE, Univ. Bordeaux, Biologie du Fruit et Pathologie, UMR 1332, Centre INRAE de Nouvelle Aquitaine Bordeaux, Villenave d’Ornon, F-33140 France; 2 Bordeaux Metabolome, MetaboHUB, PHENOME-EMPHASIS, Centre INRAE de Nouvelle Aquitaine Bordeaux, Villenave d’Ornon, F-33140 France; 3 Present Address: Laboratoire de Recherche en Sciences Végétales, UMR 5546 UPS/CNRS, Auzeville- Tolosane, F-31320 France; 4 Present Address: INRAE, Mycologie et Sécurité des Aliments, UR 1264, Centre INRAE de Nouvelle Aquitaine Bordeaux, Villenave d’Ornon, F-33140 France; 5 Max Planck Institute of Molecular Plant Physiology, am Muehlenberg 14476, Potsdam-Golm, Germany; 6 Present Address: INRAE, UR1268 BIA, Centre INRAE Pays de Loire – Nantes, Nantes, F-44000 France; 7 Present address: INRAE, BIBS Facility, Centre INRAE Pays de Loire – Nantes, Nantes, F-44000 France

**Keywords:** Fruit, LC-MS, Metabolism regulation, Proton NMR, Omics, *Solanum lycopersicum*

## Abstract

**Background:**

The composition of ripe fruits depends on various metabolites which content evolves greatly throughout fruit development and may be influenced by the environment. The corresponding metabolism regulations have been widely described in tomato during fruit growth and ripening. However, the regulation of other metabolites that do not show large changes in content have scarcely been studied.

**Results:**

We analysed the metabolites of tomato fruits collected on different trusses during fruit development, using complementary analytical strategies. We identified the 22 least variable metabolites, based on their coefficients of variation. We first verified that they had a limited functional link with the least variable proteins and transcripts. We then posited that metabolite contents could be stabilized through complex regulations and combined their data with the quantitative proteome or transcriptome data, using sparse partial-least-square analyses. This showed shared regulations between several metabolites, which interestingly remained linked to early fruit development. We also examined regulations in specific metabolites using correlations with individual proteins and transcripts, which revealed that a stable metabolite does not always correlate with proteins and transcripts of its known related pathways.

**Conclusions:**

The regulation of the least variable metabolites was then interpreted regarding their roles as hubs in metabolic pathways or as signalling molecules.

**Supplementary Information:**

The online version contains supplementary material available at 10.1186/s12870-023-04370-0.

## Background

Fruit metabolites are key players in both fleshy fruit development and human nutrition. Many studies have described metabolite changes during fruit development [1]. Significant changes have been described in soluble sugars, organic acids, amino acids and in several families of specialized metabolites, including phenolics and isoprenoids. Multi-omic approaches have given clues to the regulation of such metabolite changes, especially during ripening in tomatoes [2–4], grape berries [5, 6] and strawberries [7].

The environment and microenvironment experienced by the fruit during its development may also impact its metabolism and final composition [8], as observed in grape berry bunches [9] and tomato trusses [10], in relation with the notion of metabolism plasticity.

To compare fruit developmental stages or environmental conditions within and especially between experiments, absolute quantification is ideal. However, omics absolute quantification data remain rare despite their crucial interest for meta-analyses and metabolic model parameterization. Relative quantification is widely used in metabolomics, although absolute quantification data can be acquired with dedicated protocols [11]. In shotgun proteomics, absolute quantification is possible yet uncommon [12, 13]. In transcriptomics, absolute quantification data are accessible by spiking of internal standards in plant extracts at the beginning of the RNA purification process [14]. Although obtaining such omics data requires more effort, it allows rigorous comparisons between experiments of a given species and between fruit species.

Transcripts, proteins and metabolites that are differentially expressed or accumulated during development have usually been studied more than those showing more stable patterns. However, the most stable ones may play crucial roles. In the 90^s^, the notion of ‘house-keeping’ genes aroused special interest in RT-PCR data normalization [15, 16] and has remained in use ever since [17–19]. House-keeping proteins were identified and used in a similar way and with the same limitations [20]. The notion of house-keeping metabolite has rarely been mentioned or used for normalization. To our knowledge, only one study mentioned it in a fitting strategy minimizing the molecular profile difference of a group of molecules unaffected by the biological treatment in an iterative algorithm proposed for LC-MS data normalization [21]. However, metabolites with a stable content in several conditions might be sentinels or hubs crucial for cell functioning throughout development and may be submitted to fine-tuning.

The objectives of the present study were (i) to identify the metabolites that varied the least from a tomato fruit metabolome dataset obtained from a range of analytical strategies (most of them providing absolute quantification data), (ii) to verify whether these metabolites have a functional link with the least variable proteins and transcripts measured in the same samples and in datasets from a repository, and (iii) to combine these metabolite data with quantitative proteome and transcriptome data, to highlight the shared and specific regulations of metabolite contents.

## Results

### All metabolomic strategies contributed to differentiate fruit stages but 22 metabolites were more stable

**Fig. 1:**
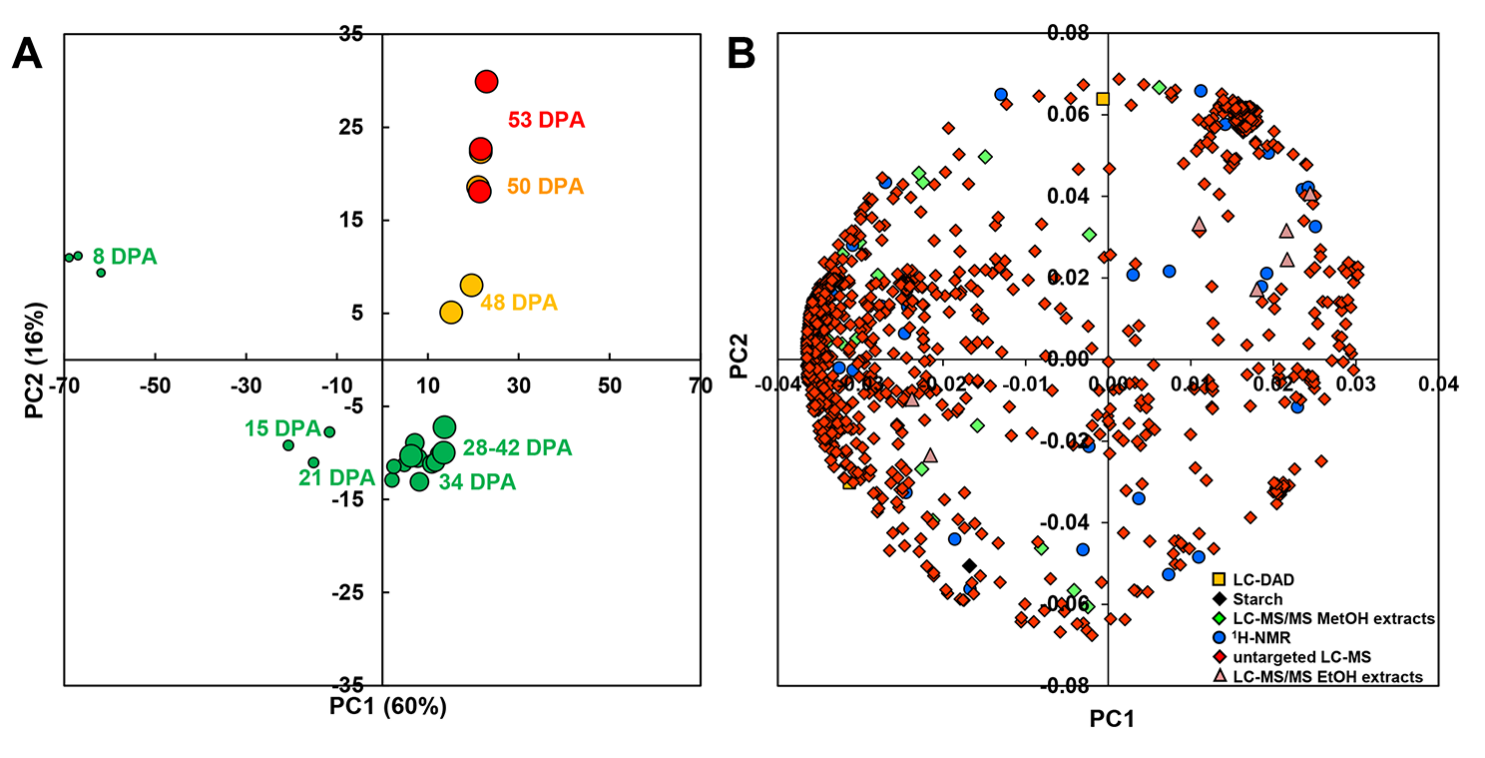
PCA of the metabolome data. The data comprised 1,243 variables expressed on a FW basis and determined in 25 samples, measured using ^1^ H-NMR, untargeted LC-MS, targeted LC-MS/MS, LC-DAD or enzymatic analyses in pericarp at nine stages of tomato fruit development in three trusses and expressed on a FW basis. (**A**) PC1xPC2 scores plot, the symbol size indicates fruit size changes. (**B**) PC1xPC2 loadings plots, the symbol shape and colour depend on the analytical strategy. See Additional file 1 for metabolite annotation on the loadings plot

The metabolome data comprised 1,243 variables from five analytical strategies (deposited in https://entrepot.recherche.data.gouv.fr): enzymatic analysis of starch, proton nuclear magnetic resonance (^1^ H-NMR) profiling of major polar compounds, targeted liquid chromatography coupled to tandem mass spectrometry (LC-MS/MS) of organic or amino acids and intermediaries of central metabolism, liquid chromatography coupled with diode array detection (LC-DAD) of isoprenoids, untargeted liquid chromatography coupled to quadrupole time-of-flight mass spectrometry (LC-QTOF-MS). To obtain an overview of the metabolome data, a principal component analysis (PCA) was performed on these variables. The scores plot (Fig. 1A) showed that the trusses of one stage tended to cluster: the differences within a cluster were lower than or similar to the differences between two successive stages of development. The first two principal components (PC) accounting for 76% of total variability separated the fruit stages. PC1 clearly separated 8 DPA and 15 DPA samples on its negative side from all the other samples on its positive side. Ripening from 42 to 53 days post-anthesis (DPA) corresponded to an upward move along PC2. The comparison of the scores plot and the loadings plot (Fig. 1B) showed that all analytical strategies contributed to stage separation based on composition. The youngest stage was characterized by a higher content in most variables determined by LC-QTOF-MS, most of them being specialized metabolites. A detailed annotation of the loadings plot (Additional file 1) showed higher sucrose, quinate, glucose-6-phosphate, chlorophylls, and chlorogenate contents at the earliest stage. The 15 to 34 DPA stages were characterized by the highest contents in ribulose-1,5-bisphosphate and sedoheptulose-bisphosphate. The 53 DPA ripe stage was characterized by the highest contents in β-carotene and glutamate as expected, but also in glutamine, galactose, glucose-1-P, caffeate and naringenin. We verified that the pericarp water content had little impact on this overview of metabolome data by performing a PCA on the same data expressed on a DW basis (Additional file 2) which showed the same tendencies for the scores and the loadings plots.

To measure metabolite variability, we calculated coefficients of variation (CVs) between trusses and between stages (Additional file 3). The mean CVs calculated between trusses ranged from 4 to 66% (Additional file 4A). The CVs calculated between stages ranged from 13 to 275% (Additional file 4B). We selected 55 variables with values below 25% for the mean CV calculated between trusses and for the CV calculated between stages (Additional file 3, Additional file 7): two variables measured using targeted LC-MS/MS, eight variables measured using ^1^ H-NMR and 45 MS-based metabolite signatures obtained from untargeted LC-MS. Examination of the latter MS-signature data revealed the presence of one peptide and redundancy due to in-source fragmentation, adducts and isotopes. After these verifications, 22 metabolites or metabolite signatures were kept as most stable metabolites for further analyses: malate, dihydroxyacetone-phosphate (DHAP), glucose, fructose, mannose, rhamnose, γ-aminobutyrate (GABA), phenylalanine, tyrosine, an unknown NMR-based metabolite signature (R_unkD5.56) and 12 MS-based metabolite signatures. The unknown NMR-based signature at 5.56 ppm had a too small intensity to observe correlations in HSQC. Its coupling in JRES was about 8–9 Hz, i.e. close to the coupling of the beta anomeric protons of a hexose moiety. In COSY, its doublet correlated with a massive hidden under the sugar signals at 3.56 ppm, but no other information could be obtained for its annotation. A tentative annotation of the MS-based metabolite signatures suggested several raw molecular formulae without further identification with a Metabolomics Standards Initiative (MSI) level 2 or 1, except for Q_M206T391 identified as a tryptophan signature (Additional file 7). To illustrate the pattern difference between most stable metabolites and other metabolites, the mean contents of most stable metabolites and five changing metabolites from different biochemical families (soluble sugars, organic acids, amino acids, intermediaries of primary metabolism and specialized metabolites) at all stages of development are shown in Additional file 6.

### Several highly stable proteins but only a few highly stable transcripts are involved in metabolism

First, we calculated the mean CV of proteins or transcripts per functional category (Additional file 5) to find and compare the categories with the lowest CV values (Wilcoxon test, corrected *P* < 0.01). For proteins, the functional categories with the three lowest means for CVs between stages of development (Additional file 5A) were BIN14 (DNA damage response), BIN1 (photosynthesis) and BIN25 (nutrient uptake). For instance, BIN14 CV between stages was significantly lower than BIN12 (chromatin organisation) one (Additional file 5B). The three functional categories with the lowest means for CVs between trusses (Additional file 5C) were BIN25 (nutrient uptake), BIN10 (redox homeostasis) and BIN2 (cellular respiration). For instance, BIN25 (Additional file 5D) had a significantly lower CV between trusses than BIN9 (secondary metabolism), BIN11 (phytohormone action), BIN21 (cell wall organisation) or BIN22 (vesicle trafficking). For transcripts, the functional categories with the three lowest means for CVs between stages of development (Additional file 5E) were BIN16 (RNA processing), BIN22 (vesicle trafficking) and BIN2 (cellular respiration). For instance, BIN16 had a significantly lower CV between stages than all BINs, except BIN2 (cellular respiration), BIN14 (DNA damage response), BIN22 (vesicle trafficking), BIN23 (protein translocation) and BIN25 (nutrient uptake) (Additional file 5F). The three functional categories with the lowest means for CVs between trusses (Additional file 5G) were BIN17 (protein biosynthesis), BIN2 (cellular respiration) and BIN23 (protein translocation). For instance, BIN17 had a significantly lower CV between trusses than all categories (Additional file 5H), except BIN2 (cellular respiration). Overall, except for respiration, redox homeostasis and polyamine biosynthesis, the functional categories involved in metabolism were not among the categories with the lowest CVs.

In line with the hypothesis of the ‘steady state’ of both metabolite contents and metabolic fluxes, we searched for the proteome and transcriptome variables that were also the most stable, to verify any putative functional link with the most stable metabolites. Although we used the same 25% CV threshold for proteins, we increased this threshold to 40% for the transcriptome data to be able to select more than 50 variables. We selected 120 proteins with values below 25% for the mean CV calculated between trusses and for the CV calculated between stage means. Sixty-four of these most stable proteins were annotated (Table 1). An enrichment analysis within this protein group did not reveal any overrepresentation or underrepresentation (Fisher’s tests with false discovery rate (FDR) correction, *P* > 0.05). Nineteen proteins were involved in primary metabolism: five in carbohydrate metabolism, four in amino acid metabolism, three in lipid metabolism, two in cellular respiration, and five in photosynthesis. Three proteins were involved in other metabolic pathways. Five proteins were involved in solute transport. Eight proteins were involved in protein homeostasis, and four in multi-process regulations. Among the proteins involved in central metabolism, two PEP-carboxylases and a cytosolic NADP-dependent malic enzyme may contribute to malate stability, and a cytosolic fructose-1,6-bisphosphatase, an ATP-dependent phosphofructokinase and an aldose 6-phosphate reductase may contribute to fructose stability. We verified that the contents of the six latter proteins were close neither to the detection limit nor to signal saturation.

**Table 1:** List of most stable proteins. Proteins in tomato fruit with a coefficient of variation (CV) between stages of development or a coefficient of variation between trusses below 25%

Proteins	CV between stages	CV between trusses	Annotation in Mercator	BINCODE	Functional category in Mercator
P_Solyc02g014150.2	23.26	13.32	photosystem II stability/assembly factor	1.1.1.3.5	Photosynthesis.photophosphorylation
P_Solyc04g082250.2	18.09	12.19	protease	1.1.1.4.1	Photosynthesis.photophosphorylation
P_Solyc01g100650.2	17.68	18.33	non-photochemical quenching regulatory protein (SOQ1)	1.1.1.5.3.2.2	Photosynthesis.photophosphorylation
P_Solyc12g014250.1	24.55	13.86	PEP carboxylase	1.4.1.1	Photosynthesis.CAM/C4 photosynthesis
P_Solyc05g056270.2	15.27	14.89	PEP carboxylase	1.4.1.1	Photosynthesis.CAM/C4 photosynthesis
P_Solyc09g010560.1	19.07	14.81	oxoprolinase	10.3.2.2	Redox homeostasis.glutathione-based redox regulation
P_Solyc11g011250.1	24.89	12.20	dehydroascorbate reductase (DHAR)	10.5.3	Redox homeostasis.ascorbate-based redox regulation
P_Solyc08g068570.2	9.49	17.04	tocopherol cyclase (VTE1/TC)	10.6.5	Redox homeostasis.tocopherol biosynthesis
P_Solyc11g064890.1	21.85	20.22	brassinosteroid signalling protein kinase (BSK)	11.3.2.1.4	Phytohormone action.brassinosteroid
P_Solyc03g121700.2	18.67	20.73	histone chaperone (NAP)	12.2.6	Chromatin organisation.histone chaperone activities
P_Solyc10g084210.1	22.08	11.49	Qc-SNARE component SYP71 of SNARE cell-plate vesicle fusion complex	13.4.2.3.3	Cell cycle organisation.cytokinesis
P_Solyc01g095200.2	17.49	15.17	ER-associated protein (Reticulon)	13.4.5.1	Cell cycle organisation.cytokinesis
P_Solyc03g120720.2	17.88	13.64	protein disulfide isomerase (PDI-L)	18.12.1.4	Protein modification.cysteine disulfide formation
P_Solyc03g123540.2	22.39	19.77	class-C-III small heat-shock-responsive protein	19.1.8.3	Protein homeostasis.protein quality control
P_Solyc09g011450.2	24.13	11.93	26 S proteasome regulator (PTRE1)	19.2.5.3.1	Protein homeostasis.ubiquitin-proteasome system
P_Solyc03g033620.2	17.60	16.20	S28-class serine carboxypeptidase	19.4.2.7	Protein homeostasis.proteolysis
P_Solyc01g100520.2	22.60	21.78	proteolytic core component ClpP1/3–6 of chloroplast Clp-type protease complex	19.4.2.9.1	Protein homeostasis.proteolysis
P_Solyc07g051850.2	21.07	14.47	pepsin-type protease	19.4.3.1	Protein homeostasis.proteolysis
P_Solyc03g111180.2	23.03	14.94	M18-class aspartyl aminopeptidase (DAP)	19.4.5.6.4	Protein homeostasis.proteolysis
P_Solyc08g062630.2	21.11	12.67	M1 neutral/aromatic-hydroxyl amino acid aminopeptidase	19.4.5.6.5	Protein homeostasis.proteolysis
P_Solyc04g079440.2	22.41	14.47	serpin protease inhibitor	19.4.6.1	Protein homeostasis.proteolysis
P_Solyc03g111010.2	13.20	13.91	NAD-dependent glyceraldehyde 3-phosphate dehydrogenase	2.1.1.4.1	Cellular respiration.glycolysis
P_Solyc02g081400.2	15.37	15.83	glutathione-independent glyoxalase (GLY-III)	2.1.2.3	Cellular respiration.glycolysis
P_Solyc01g096780.2	22.11	16.82	inner nuclear envelope component Cter-SUN of SUN-WIP cytoskeleton-nucleoskeleton-linker complex	20.4.2.1.1	Cytoskeleton organisation.nuclear dynamics
P_Solyc03g123630.2	11.16	14.19	pectin methylesterase	21.3.1.2.1	Cell wall organisation.pectin
P_Solyc04g072850.2	14.98	17.50	bifunctional alpha-L-arabinofuranosidase and beta-D-xylosidase (BXL)	21.3.2.2.4.2	Cell wall organisation.pectin
P_Solyc02g071170.2	13.92	24.72	subunit zeta of cargo adaptor F-subcomplex	22.2.1.1.4.4	Vesicle trafficking.Golgi-ER retrograde trafficking
P_Solyc08g065900.2	24.75	12.78	component VPS32/SNF7 of ESCRT-III complex	22.4.1.3.2	Vesicle trafficking.endocytic trafficking
P_Solyc12g089340.1	14.54	15.15	component VPS35 of Retromer protein recycling complex	22.4.2.1.1	Vesicle trafficking.endocytic trafficking
P_Solyc12g096550.1	17.05	13.57	component Tic55 of inner envelope TIC translocation system	23.1.3.5.2	Protein translocation.chloroplast
P_Solyc03g082940.2	14.58	13.23	nucleocytoplasmic import karyopherin (IMB1)	23.5.1.2.2	Protein translocation.nucleus
P_Solyc06g052030.2	16.13	16.73	nucleocytoplasmic import karyopherin (IMB1)	23.5.1.2.2	Protein translocation.nucleus
P_Solyc06g082120.2	23.69	14.39	Ran-activation accessory protein (RanBP1)	23.5.1.5	Protein translocation.nucleus
P_Solyc01g110120.2	16.13	15.51	subunit a of V-type ATPase membrane V0 subcomplex	24.1.1.1.1	Solute transport.primary active transport
P_Solyc10g081530.1	19.64	16.99	subunit d of V-type ATPase membrane V0 subcomplex	24.1.1.1.3	Solute transport.primary active transport
P_Solyc07g053830.2	23.01	14.95	solute transporter (MTCC)	24.2.13	Solute transport.carrier-mediated transport
P_Solyc08g081190.2	23.41	16.04	plasma membrane intrinsic protein (PIP)	24.3.1.2	Solute transport.channels
P_Solyc11g069430.1	23.54	12.53	plasma membrane intrinsic protein (PIP)	24.3.1.2	Solute transport.channels
P_Solyc07g032740.2	23.24	12.40	aspartate aminotransferase	25.1.6	Nutrient uptake.nitrogen assimilation
P_Solyc09g098150.2	12.78	13.08	programmed cell death metacaspase-like regulator (MCP2)	27.2.4.3	Multi-process regulation.Programmed Cell Death (PCD) system
P_Solyc08g015630.2	21.47	14.43	inositol trisphosphate kinase (ITPK4)	27.5.1.5.4	Multi-process regulation.phosphatidylinositol and inositol phosphate system
P_Solyc05g052760.2	18.13	24.14	phosphatidylinositol phospholipase C (PI-PLC)	27.5.2.8	Multi-process regulation.phosphatidylinositol and inositol phosphate system
P_Solyc12g055830.1	24.00	16.76	cytosolic pyrophosphatase	27.6.1	Multi-process regulation.pyrophosphate homeostasis
P_Solyc12g056530.1	20.12	15.85	cytosolic fructose-1,6-bisphosphatase	3.1.2.3	Carbohydrate metabolism.sucrose metabolism
P_Solyc11g010450.1	24.42	16.85	ATP-dependent phosphofructokinase	3.12.1	Carbohydrate metabolism.plastidial glycolysis
P_Solyc01g110450.2	23.35	14.18	aldose 6-phosphate reductase	3.5.1	Carbohydrate metabolism.sorbitol metabolism
P_Solyc02g093830.2	19.36	13.31	glucose-6-phosphate dehydrogenase	3.9.1.1	Carbohydrate metabolism.oxidative pentose phosphate pathway
P_Solyc06g053200.2	11.53	11.58	6-phosphogluconolactonase	3.9.1.2	Carbohydrate metabolism.oxidative pentose phosphate pathway
P_Solyc11g068730.1	17.38	15.34	nitrilase	30.1.2.5	Clade-specific metabolism.Brassicaceae
P_Solyc08g076990.2	9.85	11.82	N2-acetylornithine deacetylase	4.1.1.1.1.6	Amino acid metabolism.biosynthesis
P_Solyc02g068640.2	20.29	12.75	pyrroline-5-carboxylate reductase	4.1.1.1.4.1.2	Amino acid metabolism.biosynthesis
P_Solyc02g071890.2	12.62	19.77	histidinol dehydrogenase	4.1.1.2.9	Amino acid metabolism.biosynthesis
P_Solyc01g107550.2	24.46	13.50	methylthioribose kinase (MTK)	4.1.2.2.6.4.2	Amino acid metabolism.biosynthesis
P_Solyc05g050120.2	23.91	13.02	cytosolic NADP-dependent malic enzyme	5.1.1.4	Lipid metabolism.fatty acid biosynthesis
P_Solyc10g076600.1	22.71	22.12	acyl CoA oxidase (ACX)	5.7.3.2.1	Lipid metabolism.lipid degradation
P_Solyc07g045290.2	15.10	15.87	long-chain acyl-CoA synthetase (LACS9)	5.8.2.5	Lipid metabolism.lipid trafficking
P_Solyc01g099090.2	20.83	13.57	mannosylglycoprotein endo-beta-mannosidase	50.3.2	Enzyme classification.EC_3 hydrolases
P_Solyc06g068860.2	22.34	12.86	alpha-mannosidase	50.3.2	Enzyme classification.EC_3 hydrolases
P_Solyc02g062970.2	19.14	13.64	aminopeptidase	50.3.4	Enzyme classification.EC_3 hydrolases
P_Solyc01g112280.2	21.68	12.73	peptidase M20/M25/M40 family protein	50.3.5	Enzyme classification.EC_3 hydrolases
P_Solyc11g012970.1	19.37	21.82	peptidase M20/M25/M40 family protein	50.3.5	Enzyme classification.EC_3 hydrolases
P_Solyc10g047630.1	20.03	14.57	beta-ureidopropionase	6.2.4.5	Nucleotide metabolism.pyrimidines
P_Solyc02g079100.2	20.47	13.64	riboflavin kinase	7.10.9	Coenzyme metabolism.FMN/FAD biosynthesis
P_Solyc01g087260.2	15.37	14.03	carotenoid cleavage dioxygenase (CCD1)	9.1.6.3.1	Secondary metabolism.terpenoids

Mean of nine stages for the coefficients of variation calculated between trusses per stage, and coefficient of variation between stages calculated from stage means. Only annotated proteins are kept

We selected 87 transcripts that had values below 40% for the mean CV calculated between trusses and for the CV calculated between stages. Among these transcripts, 40 were annotated (Table 2). An enrichment analysis within this transcript group did not reveal any overrepresentation or underrepresentation (Fisher’s tests with FDR correction, *P* > 0.05). Fewer transcripts than proteins were linked to metabolism. Only two of the most stable transcripts were directly involved in metabolism: a protein involved in lipid degradation and another involved in nucleotide metabolism. Two transcripts were involved in solute transport, six in transcriptional regulation, five in redox homeostasis, five in protein homeostasis and one in protein translocation.

**Table 2:** List of most stable transcripts. Transcripts in tomato fruit with a coefficient of variation (CV) between stages of development or a coefficient of variation between trusses below 40%

Transcript	CV between stages	CV between trusses	Annotation in Mercator	BINCODE	Functional category in Mercator
T_Solyc01g081270.2	36.68	28.13	glutathione S-transferase	10.3.3.3	Redox homeostasis.glutathione-based redox regulation
T_Solyc01g081310.2	33.61	25.32	glutathione S-transferase	10.3.3.3	Redox homeostasis.glutathione-based redox regulation
T_Solyc09g011550.2	37.82	38.40	glutathione S-transferase	10.3.3.3	Redox homeostasis.glutathione-based redox regulation
T_Solyc02g079960.2	30.49	13.08	H-type thioredoxin	10.4.3.2	Redox homeostasis.thiol-based redox regulation
T_Solyc09g007270.2	25.69	34.29	ascorbate peroxidase (APX)	10.5.1	Redox homeostasis.ascorbate-based redox regulation
T_Solyc07g065860.2	35.87	31.79	RGF-peptide receptor (RGFR)	11.10.1.8.2	Phytohormone action.signalling peptides
T_Solyc06g062690.2	34.87	19.64	histone chaperone (NAP)	12.2.6	Chromatin organisation.histone chaperone activities
T_Solyc04g005250.2	32.60	26.04	de novo DNA methylase (DRM)	12.5.1.11	Chromatin organisation.DNA methylation
T_Solyc04g007330.1	39.48	30.23	meiotic recombination homolog pairing factor (ASY1)	13.3.5.1.4	Cell cycle organisation.mitosis and meiosis
T_Solyc12g044900.1	39.52	39.83	ER tubulae formation factor (RHD3/RL)	13.4.5.2	Cell cycle organisation.cytokinesis
T_Solyc03g026020.2	32.98	13.67	transcription factor (HSF)	15.5.13	RNA biosynthesis.transcriptional regulation
T_Solyc07g063420.2	34.38	21.08	transcription factor (NAC)	15.5.17	RNA biosynthesis.transcriptional regulation
T_Solyc01g057910.2	35.51	33.40	transcription factor (MYB)	15.5.2.1	RNA biosynthesis.transcriptional regulation
T_Solyc06g053220.2	37.44	33.50	transcription factor (HD-ZIP I/II)	15.5.3.1	RNA biosynthesis.transcriptional regulation
T_Solyc06g061240.2	32.43	17.21	transcription factor (PLATZ)	15.5.41	RNA biosynthesis.transcriptional regulation
T_Solyc05g006040.2	33.17	33.73	component PRIN2 of plastid-encoded RNA polymerase	15.6.1.2.3.9	RNA biosynthesis.organelle machinery
T_Solyc07g064620.1	39.78	17.97	assembly factor (eIF1) of eIF1	17.4.1.1.1	Protein biosynthesis.translation initiation
T_Solyc05g009010.1	38.82	25.15	protein kinase (LRK10-1-like)	18.4.1.20	Protein modification.phosphorylation
T_Solyc10g012240.2	38.24	15.64	ubiquitin-conjugating component GID3 of GID ubiquitination complex	19.2.1.3.1.3	Protein homeostasis.ubiquitin-proteasome system
T_Solyc05g012560.1	24.76	25.73	RING-HC-class E3 ligase	19.2.2.1.4.3.2	Protein homeostasis.ubiquitin-proteasome system
T_Solyc04g011430.2	28.89	13.41	component Ubc13 of Ubc13-Uev1 conjugating E2 complex	19.2.2.1.5.1.1	Protein homeostasis.ubiquitin-proteasome system
T_Solyc04g079970.2	34.18	15.24	RUB conjugation E2 protein (RCE1)	19.2.2.3.3	Protein homeostasis.ubiquitin-proteasome system
T_Solyc04g079480.2	24.12	35.22	serpin protease inhibitor	19.4.6.1	Protein homeostasis.proteolysis
T_Solyc09g072600.1	36.44	21.35	actin-depolymerizing factor	20.2.2.8	Cytoskeleton organisation.microfilament network
T_Solyc09g072590.2	31.74	17.14	actin-depolymerizing factor	20.2.2.8	Cytoskeleton organisation.microfilament network
T_Solyc07g064180.2	28.00	24.26	pectin methylesterase	21.3.1.2.1	Cell wall organisation.pectin.homogalacturonan
T_Solyc06g076450.2	35.59	24.51	A-class RAB GTPase	22.3.4.1.1	Vesicle trafficking.post-Golgi trafficking
T_Solyc02g093330.2	34.05	39.99	nucleoporin of nuclear pore complex (NUP98)	23.5.1.1.6.1	Protein translocation.nucleus
T_Solyc11g065820.1	39.02	34.99	metabolite transporter (DTX)	24.2.4.1.1	Solute transport.carrier-mediated transport
T_Solyc05g051220.2	39.27	26.73	voltage-gated potassium cation channel (AKT/SKOR/GORK)	24.3.2.2	Solute transport.channels
T_Solyc08g060920.2	25.69	19.44	phosphate signalling regulatory protein (SPX)	25.3.1.2	Nutrient uptake.phosphorus assimilation
T_Solyc12g056650.1	39.32	36.55	zeitlupe-mediated photoperception regulator protein (GIGANTEA)	26.1.2.3.2	External stimuli response.light
T_Solyc02g083280.2	22.95	20.25	arsenate reductase (HAC)	26.7.2.2	External stimuli response.toxic compounds
T_Solyc09g059430.2	38.35	28.01	effector-triggered immunity RPM1-interacting factor (RIN4)	26.9.2.2.2	External stimuli response.pathogen
T_Solyc07g017510.2	33.15	29.12	phosphatidylinositol 3-phosphate 5-kinase (FAB1)	27.5.1.4.4	Multi-process regulation.phosphatidylinositol and inositol phosphate system
T_Solyc06g053670.1	29.54	18.42	dodecenoyl-CoA isomerase	5.7.3.5.1	Lipid metabolism.lipid degradation
T_Solyc05g008290.2	39.48	22.45	multicopper oxidase LPR1	50.1.10	Enzyme classification.EC_1 oxidoreductases
T_Solyc01g096280.1	39.17	39.57	cytochrome P450 78A3	50.1.13	Enzyme classification.EC_1 oxidoreductases
T_Solyc12g009420.1	36.72	37.64	polygalacturonase QRT2	50.3.2	Enzyme classification.EC_3 hydrolases
T_Solyc05g005600.1	28.32	38.72	deoxyguanidine triphosphatase	6.3.3.3	Nucleotide metabolism.deoxynucleotides

Mean of nine stages for the coefficients of variation calculated between trusses per stage, and coefficient of variation between stages calculated from stage means. Only annotated transcripts are kept

### Several proteins and transcripts covary with sets of the most stable metabolites

**Fig. 2:**
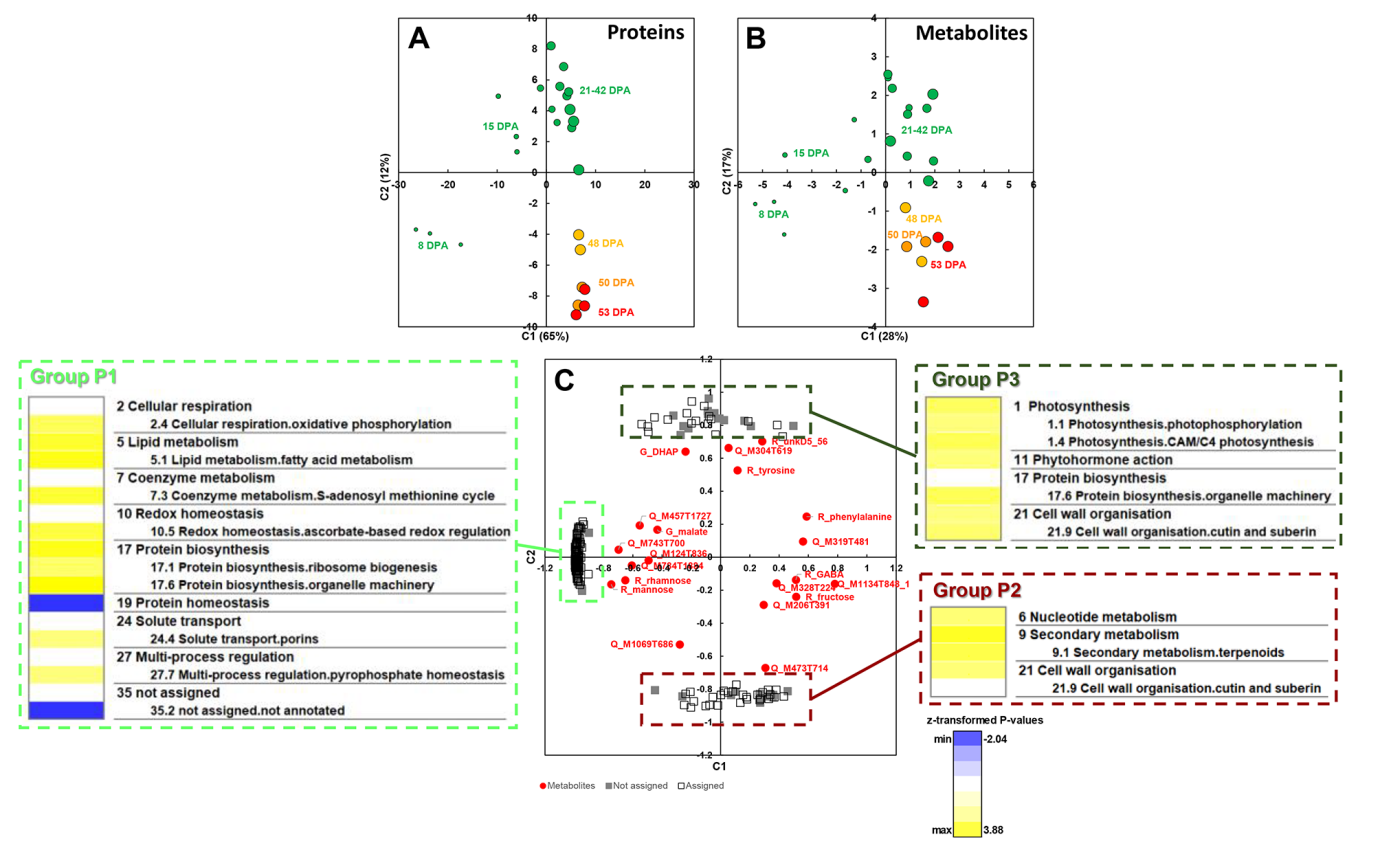
sPLS analyses of 22 most stable metabolites with proteome data. The sparse approach was parameterized to select 10 times more proteome variables than the metabolite ones. (**A**) Scores plot of the selected proteome data. (**B**) Scores plot of most stable metabolome data. (**C**) Loadings plot combining the proteome and metabolome data. For each protein group (P1, P2, P3), the PageMan functional categories of genes are displayed. Fisher’s test was used to identify functional categories over-represented or under-represented within a protein group compared to the entire 2,282 protein set. Coloured boxes indicate statistically-significant groups (Fisher’s test p-value < 0.05). The colour scale represents z-transformed p-values, with yellow shades indicating a trend within the group for over-representation relative to the 2,282 protein set, and blue shades under-representation relative to the 2,282 protein set. Text on the right indicates PageMan annotation of protein classes

In line with the hypothesis that the lowest variability in the contents of the most stable metabolites resulted from complex metabolic flux regulations, we searched for common trends between several metabolites and proteins or transcripts by using a multiblock sparse partial-least-square analysis (sPLS) approach for proteome and transcriptome data separately. This approach was used to explore and visualize the links between metabolites and proteins (or transcripts) by maximizing the covariance between the latent variables of the two data blocks. It also allowed the selection of a given number of variables with its sparse mode. To focus on important variables and facilitate their biological interpretation, we selected 10 times more proteome or transcriptome variables than metabolites. First, an sPLS analysis combining the most stable metabolites and proteome data allowed the selection of 210 protein variables covarying with these metabolites (Fig. 2). Scores plots (Fig. 2A-B) showed a clear separation of the first two stages of development from all other stages along component 1, and a clear separation of the last two stages of development from all other stages along component 2. Loadings were combined on the same plot to highlight the co-regulations of metabolites and proteins. The common loadings plot of the most stable metabolites and proteins highlighted four groups of variables, three of them with a similar trend for several metabolites and several proteins (Fig. 2C). One hundred and forty proteins (protein Group P1) tended to covary (loading value over 0.6) with mannose, rhamnose, Q_M743T700 and Q_M784T1884. An enrichment analysis (Fig. 2C) within this protein group showed an overrepresentation of several categories, including BIN2.4 (cellular respiration.oxidative phosphorylation), BIN5.1 (lipid metabolism.fatty acid metabolism), BIN7.3 (coenzyme metabolism.S-adenosyl methionine (SAM) cycle), BIN10.5 (redox homeostasis.ascorbate-based redox regulation), BIN17.1 (protein biosynthesis.ribosome biogenesis), BIN17.6 (protein biosynthesis.organelle machinery), and an underrepresentation of BIN19 (protein homeostasis) and unannotated proteins. Forty proteins (protein Group P2) tended to covary with Q_M473T714. An enrichment analysis (Fig. 2C) within this protein group showed an overrepresentation of BIN6 (nucleotide metabolism), BIN9.1 (secondary metabolism.terpenoids) and BIN21 (cell wall organisation) category. Thirty proteins (protein Group P3) tended to covary with DHAP, Q_M304T619 and R_unkD5.56. The enrichment analysis in the latter protein group (Fig. 2C) showed an overrepresentation of several categories including BIN1.1 (photosynthesis.photophosphorylation), BIN1.4 (photosynthesis.CAM/C4 photosynthesis), BIN11 (phytohormone action), BIN17.6 (protein biosynthesis.organelle machinery) and BIN21.9 (cell wall organisation.cutin and suberin).

**Fig. 3:**
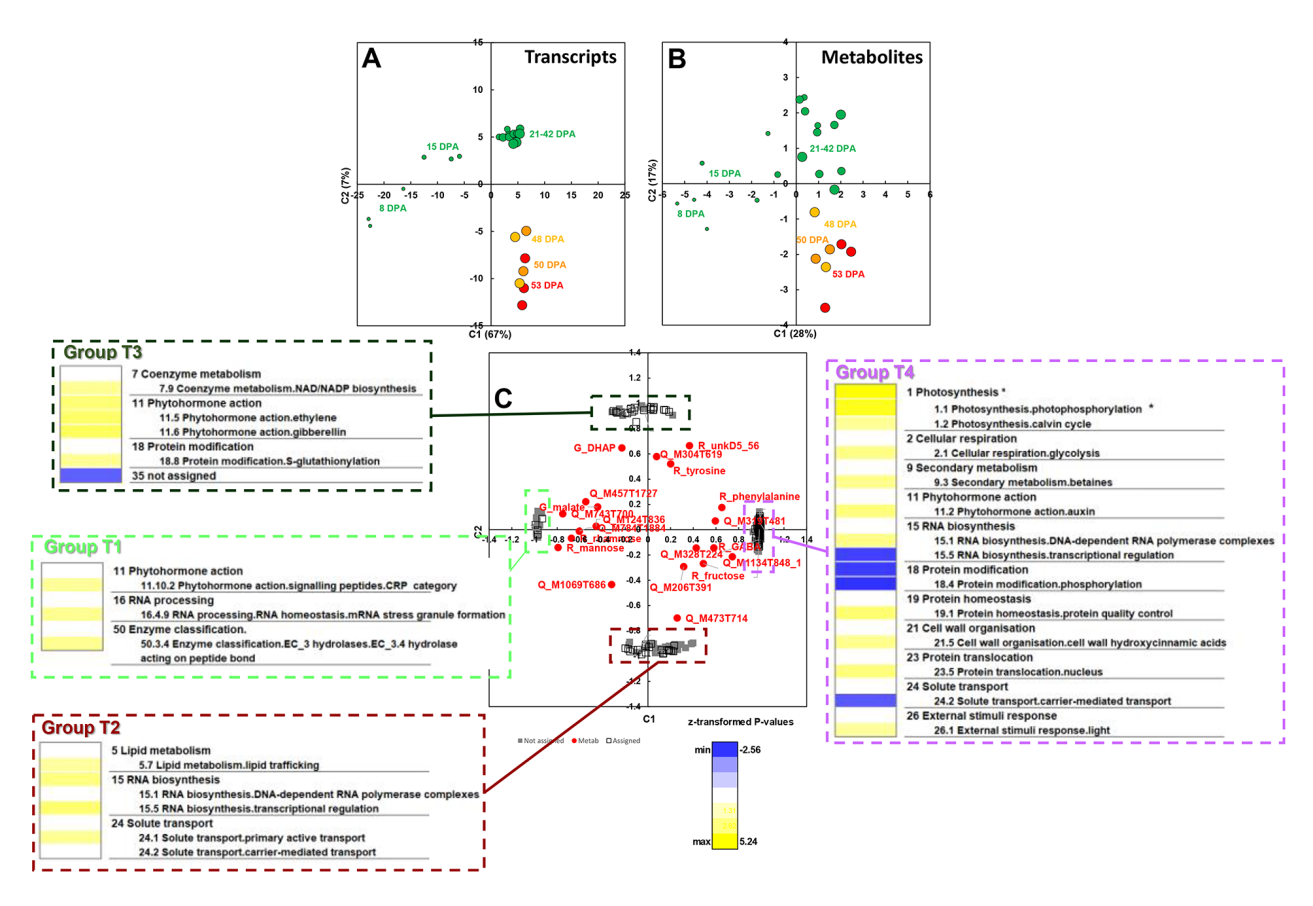
sPLS analyses of 22 most stable metabolites with transcriptome data. The sparse approach was parameterized to select 10 times more transcriptome variables than the metabolite ones. (**A**) Scores plot of the selected transcriptome data. (**B**) Scores plot of most stable metabolome data. (**C**) Loadings plot combining the proteome and metabolome data. For each transcript group (T1, T2, T3, T4), the PageMan functional categories of genes are displayed. Fisher’s test was used to identify functional categories over-represented or under-represented within a protein group compared to the entire 23,631 transcript set. Coloured boxes indicate statistically-significant groups (Fisher’s test p-value < 0.05). The colour scale represents z-transformed p-values, with yellow shades indicating a trend within the group for over-representation relative to the 23,631 transcript set, and blue shades under-representation relative to the 23,631 transcript set. * indicates that the corrected Fisher’s test p-value was below 0.05

Second, an sPLS analysis combining the most stable metabolites and transcriptome data allowed the selection of 210 transcript variables covarying with these metabolites (Fig. 3). Scores plots (Fig. 3A-B) showed the same separations as in Fig. 2. The common loadings plot of the most stable metabolites and transcripts highlighted four groups of variables with a similar trend for several metabolites and proteins (Fig. 3C). Fourteen transcripts (transcript Group T1) tended to covary with mannose, rhamnose, Q_M743T700 and Q_M784T1884. An enrichment analysis (Fig. 3C) in this transcript group showed neither overrepresentation nor underrepresentation. Thirty-nine transcripts (transcript Group T2) tended to covary with Q_M473T714. An enrichment analysis (Fig. 3C) in this transcript group showed an overrepresentation of several categories, including BIN5.7 (lipid metabolism.lipid trafficking), BIN15 (RNA biosynthesis) and BIN24.1 (solute transport.primary active transport). Thirty-one transcripts (transcript Group T3) tended to covary with DHAP, Q_M304T619 and R_unkD5.56. An enrichment analysis (Fig. 3C) in this transcript group showed an overrepresentation of BIN7.9 (coenzyme metabolism.NAD/NADP biosynthesis), BIN11.5 (phytohormone action.ethylene), BIN11.6 (phytohormone action.gibberellin) and BIN18.8 (protein modification.S-glutathionylation) and an underrepresentation of unannotated transcripts. One hundred and twenty-six transcripts (transcript Group T4) tended to covary with phenylalanine and Q_M1134T848_1. An enrichment analysis (Fig. 3C) in the latter transcript group showed an overrepresentation of 11 categories, including BIN1.1 (photosynthesis.photophosphorylation), BIN1.2 (photosynthesis.calvin cycle), BIN2.1 (cellular respiration.glycolysis), BIN9.3 (secondary metabolism.betaines), BIN11.2 (phytohormone action.auxin), BIN15.1 and BIN19.1 (protein homeostasis.protein quality control), and an underrepresentation of BIN15.5 (RNA biosynthesis.transcriptional regulation), BIN18.4 (protein modification.phosphorylation) and BIN24.2 (solute transport.carrier-mediated transport).

### Several proteins and transcripts correlate with a given metabolite

To complete this approach aimed at highlighting regulations or stabilities shared between several of the most stable metabolites, we studied the links between each metabolite variable and all the variables of the other two omics datasets in an unfocused manner. To search for such trends specific to a given metabolite, we used non-linear correlations (Spearman) between that metabolite and the proteome and transcriptome datasets separately. Nine metabolites correlated with a threshold of *P* < 0.0001, with at least 10 assigned proteins or 10 assigned transcripts (Table 3): mannose, rhamnose, and metabolite signatures R_unkD5.56, Q_M124T836, Q_M319T481, Q_M473T714, Q_M743T700, Q_M1069T686 and Q_M1134T848_1. We performed an enrichment analysis of the proteins (Additional file 8) or transcripts (Additional file 9) positively or negatively correlated with a given metabolite (Fisher test, corrected *P* < 0.05).

**Table 3:** Summary of the correlation analyses between the most stable metabolites or metabolite signatures and proteins or transcripts

Variable name	Metabolite name	Proteins				Transcripts		
		Number of correlated proteins	Number of assigned correlated proteins	Ontology description of the largest bin ^a^ (number of proteins per bin)		Number of correlated transcripts	Number of assigned correlated transcripts	Ontology description of the largest bin ^a^ (number of transcripts per bin)
G_malate	malate	1	1	-		5	1	-
G_DHAP	dihydroxyacetone-phosphate	3	2	-		13	4	-
R_glucose	glucose	0	0	-		0	0	-
R_fructose	fructose	0	0	-		4	3	-
**R_mannose**	mannose	14	10	-		472	248	RNA biosynthesis (31) including RNA biosynthesis.transcriptional regulation (29)
**R_rhamnose**	rhamnose	22	17	Protein translocation (4)		170	87	Protein homeostasis (17) including Protein homeostasis.proteolysis (14)
R_GABA	GABA	6	5	-		18	9	-
R_phenylalanine	phenylalanine	0	0	-		2	0	-
R_tyrosine	tyrosine	9	5	-		15	6	-
**R_unkD5_56**		20	14	-		70	42	RNA biosynthesis (6)
**Q_M124T836**		1	1	-		40	23	RNA biosynthesis (6)
Q_M206T391		0	0	-		0	0	-
Q_M304T619		3	2	-		19	13	Solute transport (3)
**Q_M319T481**		18	14	RNA processing (3)		103	67	Cellular respiration (9)
Q_M328T224		0	0	-		1	0	-
Q_M457T1727		10	8	-		23	8	-
**Q_M473T714**		33	21	Amino acid metabolism (3), Cell wall organisation (3)		125	59	RNA biosynthesis (15) including RNA biosynthesis.transcriptional regulation (15)
**Q_M743T700**		5	2	-		832	416	RNA biosynthesis (54) including RNA biosynthesis.transcriptional regulation (48)
Q_M784T1884		1	1	-		6	3	-
**Q_M1069T686**		1	1	-		31	16	
Q_M1093T854_3		0	0	-		2	1	-
**Q_M1134T848_1**		839	704	Protein biosynthesis (134) including Protein biosynthesis.ribosome biogenesis (79)		542	281	RNA biosynthesis (37) including RNA biosynthesis.transcriptional regulation (37)

Summary by metabolite or metabolite signature with a Spearman correlation threshold of *P* < 0.0001. Metabolites correlated with this threshold with at least 10 assigned proteins or 10 assigned transcripts are in bold

^a^ Only bins containing more than two assigned proteins or transcripts are listed

For M1134T848 only, enrichments were observed for both the proteins and transcripts with the defined correlation threshold. The proteins positively correlated with M1134T848 showed an overrepresentation of BIN17 (protein biosynthesis) and six of its subcategories (BIN17.1, BIN17.1.2, BIN17.1.2.1, BIN17.1.3, BIN17.1.3.1, BIN17.6.2), and an underrepresentation of BIN19 (protein homeostasis) and one of its subcategories (BIN19.2, Additional file 8). The transcripts positively correlated with M1134T848 showed an overrepresentation of BIN24.3.1 (solute transport.channels.MIP family) and one of its subcategories (BIN24.3.1.2, Additional file 9).

The transcripts (Additional file 9) negatively correlated with mannose showed an overrepresentation of BIN3.11 (carbohydrate metabolism.fermentation) and two of its subcategories (BIN3.11.1, BIN3.11.1.1), of BIN13.2.1 (cell division.cell cycle organization.cell cycle control) and two of its subcategories (BIN13.2.1.1, BIN13.2.1.1.2), and of BIN19.4.6 (protein homeostasis.proteolysis.protease inhibitor activities) and one of its subcategories (BIN19.4.6.2). The transcripts negatively correlated with rhamnose showed an overrepresentation of BIN19.4.6 (protein homeostasis.proteolysis.protease inhibitor activities) and one of its subcategories (BIN19.4.6.2).

The transcripts positively correlated with M319T481 showed an overrepresentation of BIN2 (cellular respiration) and two of its subcategories (BIN2.4, BIN2.4.3), and of BIN10 (redox homeostasis) and two of its subcategories (BIN10.2, BIN10.0.1.3).

The transcripts negatively correlated with M743T700 showed an overrepresentation of BIN13 (cell division) and four of its subcategories (BIN13.2, BIN13.2.1, BIN13.2.1.1, BIN13.2.1.1.2), of BIN5.5.2 (lipid metabolism.phytosterol metabolism.phytosterol C4-demethylation complex), and of BIN 19.4.6 (protein homeostasis.proteolysis.protease inhibitor activities) and one of its subcategories (BIN19.4.6.2).

### The most stable metabolites do not always correlate with proteins and transcripts of their known related pathways

We then focused on the known pathways of these metabolites. We listed the proteins and transcripts linked to each metabolite’s metabolic pathways (Additional file 10) and looked for the 10 proteins and 10 transcripts with the highest absolute values of Spearman correlation coefficients (Additional file 11). For some metabolites, proteins or transcripts correlation p-values below 0.001 were rare (Table 4), yet several correlations with p-values below 0.01 remained of interest. Each list of correlated genes comprised several metabolic pathways (Additional files 10–11). This was especially true for transcripts and proteins correlated with DHAP and malate, in which several genes are involved in different metabolic pathways and link photosynthesis, respiration, carbohydrate and lipid metabolism.

**Table 4:** Proteins or transcripts of genes in known metabolic pathways implicating a given most stable metabolite (Table S4) correlated with this metabolite and selected if *P* < 0.001

Most stable metabolite	Correlated target	Target functional categories in Mercator	Target annotation in Mercator	R	*P*
**G_DHAP**	T_Solyc01g018020.1	Photosynthesis.calvin cycle	transketolase	-0.6614	3.179E-04
	T_Solyc12g008430.1	Lipid metabolism.fatty acid biosynthesis.citrate shuttle	cytosolic NADP-dependent malic enzyme	0.6193	9.628E-04
	T_Solyc10g083300.1	Carbohydrate metabolism.sucrose metabolism.degradation.invertase activities	acid beta-fructofuranosidase	0.6226	8.884E-04
**G_malate**	P_Solyc05g054370.2	Lipid metabolism.lipid degradation.fatty acid degradation.alternative beta-oxidation	monofunctionial hydroxyacyl-CoA dehydrogenase	0.6231	8.778E-04
**R_mannose**	T_Solyc09g005110.2	Carbohydrate metabolism.fermentation.acetic acid biosynthesis	pyruvate decarboxylase (PDC)	-0.7692	6.999E-06
	T_Solyc02g077240.2	Carbohydrate metabolism.fermentation.acetic acid biosynthesis	pyruvate decarboxylase (PDC)	-0.7508	1.536E-05
	T_Solyc10g076510.1	Carbohydrate metabolism.fermentation.acetic acid biosynthesis	pyruvate decarboxylase (PDC)	-0.7015	9.327E-05
	T_Solyc04g005030.2	Carbohydrate metabolism.mannose metabolism.	phosphomannomutase	-0.6762	2.070E-04
	T_Solyc07g042550.2	Carbohydrate metabolism.sucrose metabolism.degradation	sucrose synthase	-0.6738	2.218E-04
	T_Solyc12g008510.1	Carbohydrate metabolism.sucrose metabolism.degradation	hexokinase	-0.6600	3.310E-04
	T_Solyc02g081300.2	Carbohydrate metabolism.sucrose metabolism.degradation	sucrose synthase	0.6562	3.686E-04
	P_Solyc03g006860.2	Carbohydrate metabolism.sucrose metabolism.degradation	fructose kinase	0.6609	4.390E-04
	T_Solyc08g007100.2	Carbohydrate metabolism.mannose metabolism.	phosphosugar phosphatase	0.6631	3.033E-04
	T_Solyc02g086090.2	Redox homeostasis.ascorbate-based redox regulation.ascorbate metabolism.L-galactose biosynthesis pathway	phosphomannose isomerase (PMI)	0.6669	2.716E-04
	T_Solyc08g013840.2	Carbohydrate metabolism.mannose metabolism	phosphosugar phosphatase	0.7446	1.966E-05
**R_rhamnose**	P_Solyc05g005700.2	Carbohydrate metabolism.fermentation.acetic acid biosynthesis	aldehyde dehydrogenase (ALDH2B)	-0.6238	8.612E-04
**R_tyrosine**	P_Solyc03g114150.2	Protein biosynthesis.aminoacyl-tRNA synthetase activities	aldehyde dehydrogenase (ALDH2B)	0.6823	1.719E-04

Proteins (P_) or transcripts (T_) are considered as targets here. For each target, the pathway and annotation in Mercator are indicated. R is the Spearman correlation coefficient value and *P* is its corresponding p-value. For each metabolite, the 10 proteins and 10 transcripts with the highest absolute value of R were pre-selected and those with a correlation *P* < 0.001 are listed. See Additional file 11 for results before p-value filtering

Surprisingly, no gene of the known metabolic pathways of the most stable soluble sugars correlated with fructose or glucose at *P* < 0.001, and all the correlations with the corresponding listed proteins (*P* < 0.01) were negative (Additional file 11). No transcripts or proteins of known glucose pathways correlated with glucose at *P* < 0.01. Only four proteins of known pathways involving fructose correlated with fructose at *P* < 0.01. For fructose, the highest absolute value of correlation with a protein was observed with an alkaline invertase (R= -0.59, *P* = 0.0020), while the highest absolute value of correlation with a transcript was observed with a granule-bound starch amylose synthase (R = 0.47, *P* = 0.0173). Conversely, over 10 proteins and 10 transcripts of known mannose or rhamnose pathways correlated with mannose or rhamnose at *P* < 0.01, and over 10 transcripts and one protein of known mannose pathways correlated with mannose at *P* < 0.001 (Table 4). The highest absolute value of correlation with a protein was observed with a fructose kinase (R = 0.66, *P* = 0.0004), and that with a transcript was observed with a gene annotated as pyruvate decarboxylase (R=-0.77, *P* < 0.0001). Only one protein of known rhamnose pathways correlated with rhamnose at *P* < 0.001. The highest absolute value of correlation of rhamnose with a protein was observed with an aldehyde dehydrogenase (R=-0.62, *P* = 0.0009), and that with a transcript was observed with a hexokinase (R=-0.59, *P* = 0.0021). For DHAP, three transcripts of known DHAP pathways correlated at *P* < 0.001, and the highest absolute value of correlation was with a transketolase (R=-0.66, *P* = 0.0003). The highest absolute value of correlation with a protein was observed with a starch-debranching isoamylase (R = 0.62, *P* = 0.0012).

For malate, only one protein and no transcript of known malate pathways correlated at *P* < 0.001. The highest absolute value of correlation with a protein was observed with a monofunctional hydroxyacyl-CoA dehydrogenase (R = 0.62, *P* = 0.0009), and that with a transcript was observed with a malate synthase (R=-0.59, *P* = 0.0017). Concerning GABA, a most stable amino acid, the highest absolute value of correlation with a protein was observed with a diaminopimelate decarboxylase (R=-0.61, *P* = 0.0013), which catalyses the last step of lysine biosynthesis, and that with a transcript was observed with the same diaminopimelate decarboxylase (R = 0.59, *P* = 0.0018). This suggests that GABA, a product of lysine catabolism, could exert a negative feedback at the transcriptional level of lysine biosynthesis. For phenylalanine and tyrosine, more transcripts than proteins of the corresponding pathways correlated with each of the latter metabolites at *P* < 0.01. For phenylalanine, the highest absolute value of correlation observed with a protein was with a gene annotated as a phospholipase D (R = 0.58, *P* = 0.0025), while that with a transcript was observed with an acireductone dioxygenase (R = 0.55, *P* = 0.0041). Two phenylalanine ammonia lyases were correlated at 0.50 and 0.52. For tyrosine, only one protein and no transcript of known tyrosine pathways correlated at *P* < 0.001. The highest absolute value of correlation with a protein was observed with a gene annotated as an aldehyde dehydrogenase ALDH2B (R = 0.68, *P* = 0.0002), and that with a transcript was observed with an alcohol dehydrogenase (R = 0.60, *P* = 0.0017). For tryptophan, no protein and no transcript of known tryptophan pathways correlated at *P* < 0.001. The highest absolute value of correlation with a protein was observed with a gene annotated as an M1 neutral/aromatic-hydroxyl amino acid aminopeptidase (R=-0.43, *P* = 0.0337), while that with a transcript was observed with an allene oxidase synthase involved in jasmonate biosynthesis (R = 0.47, *P* = 0.0175).

## Discussion

### Several of the most stable metabolites are considered as metabolic pathway hubs

Malate, DHAP, glucose, fructose, mannose, rhamnose, GABA, phenylalanine and tyrosine appeared to be the least variable or most homeostatic, identified metabolites in tomato fruit pericarp in our experiment. Among sugars, mannose may link primary cell-wall metabolism and ascorbate biosynthesis through GDP-D-mannose epimerase [22]. Rhamnose in plants is mostly found as part of cell wall polymers or conjugated to specialized metabolites [23].

The stability of malate can be attributed to its involvement in balancing the ATP/NAD(P)H ratio in various subcellular compartments via the so-called malate valves [24]. Malate has been shown in transformant plants to play a key role in starch metabolism and ripening of tomato fruit [25]. DHAP is involved in several metabolic pathways such as photosynthesis, respiration, carbohydrate metabolism and lipid metabolism. The stability of its content in tomato pericarp may reflect the fact that it is a crossroad metabolite.

GABA plays a role in the GABA shunt related to the TCA cycle, and in the polyamine biosynthesis pathway. It can also be synthesized by a nonenzymatic reaction from proline under oxidative stress [26]. In tomato, the fact that its precursor glutamate is strongly accumulated during ripening while GABA remains relatively stable suggests that GABA homeostasis is important for fruit ripening. Besides fuelling protein synthesis, phenylalanine and tyrosine play a key role in primary and specialized metabolism as precursors of a range of specialized metabolites including phenylpropanoids. The phenylpropanoid pathway and especially its ammonia-lyases are highly regulated at the transcriptional, post-transcriptional, and post-translational levels [27]. In Arabidopsis, a metabolic crosstalk exists between cytosolic phenylalanine biosynthesis and tryptophan-dependent auxin biosynthesis [28]. A similar crosstalk may exist in fruit.

For some of these metabolites, their involvement in multiple pathways depends on their mobility toward the different plant cell compartments. This is particularly the case for malate but also for glucose and fructose for which families of channels and/or transporters have been identified [24, 29, 30]. Indeed, glucose and fructose both belong to the sucrose cycle which involves the cytosol and the vacuole in fruit cells [31]. The expansion of the latter might be largely due to the accumulation of these hexoses that predominate in osmolytes. This would also account for their great stability throughout the development of tomato fruit [32]. The existence of transporters is crucial for the subcellular partitioning of metabolites, especially in fleshy fruits [33].

### Several highly stable metabolites may also be considered as signalling molecules

In addition to being involved in different metabolic pathways, several of the most stable metabolites in the present experiment have been proposed as signalling molecules in regulatory pathways in plants: glucose [34], fructose [35] and GABA [36]. For instance, concerning the regulation of sugar metabolism, hexokinase is thought to play a key role in the uncoupling of glucose signalling from glucose metabolism in plants [37–39]. In the present study, no link was observed between glucose and a hexokinase. Only a hexokinase transcript and a fructokinase protein correlated negatively with fructose content, but with a coefficient that was not below − 0.5.

GABA has been shown to play a key role in regulating pollen tube growth and stomatal pore aperture [40], and the latter authors proposed an additional role in long-distance signalling and a possible involvement in crosstalk with hormonal signals. In fruit, GABA is involved in development [41], and the equilibrium between ethylene and GABA signalling may contribute to regulate fruit taste through the modulation of tonoplast-localized ALMT-mediated malate storage during ripening [40]. The latter authors proposed a more general role for GABA in connecting plant primary metabolism to plant physiological status, a mechanism that could be essential for fruit growth adjustment to plant fitness.

DHAP has rarely been mentioned to play a signalling role in plants. Redox control involving plastoquinone is thought to be contingent on signals related to the relative availability of trioses-P [42]. A recent study on human cells proposed DHAP as a glucose-derived signalling molecule that activates the rapamycin complex 1 (mTORC1) kinase, leading to cell growth [43]. The TOR kinase signalling pathway is determinant for plant development [44] and it contributes to regulating cell-cell transport in mature photosynthesizing leaves [45]. Whether or not the metabolic signal activation of the TOR pathway in plants involves DHAP is currently unknown. Such a signalling role might also be played by the other most stable metabolites in the present study. Indeed, mannose was shown to regulate the *Sus1* sucrose synthase gene via hexokinase-modulated mechanisms in Arabidopsis [46].

### Links between a highly stable metabolite and proteins or transcripts of its known related pathways are not always obvious

We tested whether the least variable metabolites were regulated by quite stable pathways or part of their pathways, i.e. stable proteins and transcripts, or by proteins and transcripts of their corresponding pathways exhibiting greater variations.

Overall, and with rare exceptions, the functional categories of proteins and transcripts involved in metabolism were not those with the lowest CVs. Few of the most stable proteins and the most stable transcripts involved members of the metabolic pathways of the most stable metabolites. Fewer highly stable transcripts than highly stable proteins were involved in metabolism. Only malate and fructose contents might be partly regulated by enzymes with a limited content variation between trusses and between stages of development. The other highly stable metabolites might be regulated post-transcriptionally or post-translationally, or by the effect of allosteric effectors on enzyme activities, all of which might allow rapid fine-tuning [47].

When we searched for correlations between each highly stable metabolite and transcripts or proteins in an unfocused manner, we found a limited over-representation of transcripts or proteins of related metabolic pathways. Only the transcripts negatively correlated with mannose showed an over-representation for a carbohydrate metabolism sub-category. Therefore, we searched for correlations between each highly stable metabolite and individual transcripts or proteins of its known related pathways. At a *P* < 0.001 threshold for the correlations, over 10 transcripts and one protein of known mannose pathways correlated with mannose, but no or only a few transcripts or proteins of their known metabolic pathways correlated with fructose, glucose, rhamnose, DHAP or tyrosine. Such behaviour may indicate that the fine regulation mode of the majority of the most stable metabolites in the present study changes throughout fruit development. These changes may subsume evolving metabolic priorities in line with the successive developmental stages (e.g. cell division requiring energy [32], cell expansion requiring vacuolar solutes [31]), with the possible involvement of different isoforms throughout development, as is the case for cell wall modifications and ethylene biosynthesis [48].

### Common trends of metabolite regulations are linked with early fruit development

Although the choice of metabolites was based on their limited variation during development and between trusses, they continued to show faint developmental changes. In accordance with the well-known developmental phases of tomato fruit development [2], three groups of metabolite variation profiles were obtained, fitting with the two fruit growth phases, cell division and cell expansion, and with fruit ripening. Regarding the covariations of each metabolite group with protein or transcript groups, unannotated proteins were very under-represented at early stages and transcripts at intermediary stages. This could be due to the fact that covarying protein or transcript groups at these stages have crucial functions, and are therefore well-known and well-annotated.

Mannose, rhamnose, Q_M743T700 and Q_M784T1884, with higher contents at the first stage of development, covaried with a group of proteins that were overrepresented for cellular respiration, lipid metabolism, S-adenosyl methionine (SAM) metabolism, redox homeostasis and protein biosynthesis. All these protein types are linked to cell division requiring active lipid and protein biosynthesis and a turbo respiration rate [32]. Fine-tuning of free mannose and rhamnose content at the early stages of fruit development may be crucial for cell wall remodelling, which is essential for fruit growth [49]. Whereas SAM is known to be involved in the ripening of climacteric fruit as a precursor of ethylene biosynthesis, it is also a ubiquitous methyl donor that may be crucial for polysaccharide methylation in primary cell walls [50] in the early stages of fruit development.

DHAP, Q_M304T619 and R_unkD5.56, which had higher contents during fruit expansion stages, covaried with a group of proteins overrepresented for photosynthesis, phytohormone action, protein biosynthesis and cell wall organization. DHAP as a precursor of membrane glycerolipids [51] might be crucial for cell expansion. Q_M304T619 and R_unkD5.56 could not be identified.

Phenylalanine and Q_M1134T848_1, which had higher contents in the last seven stages compared to the first two ones, showed common trends, as they covaried with a group of transcripts over-represented for photosynthesis, cellular respiration.glycolysis, secondary metabolism.betaines, phytohormone action.auxin, and protein homeostasis.protein quality control. In Arabidopsis, auxin-regulated plant growth is fine-tuned by the early stages of phenylpropanoid biosynthesis, and it has been suggested that metabolites accumulating upstream of the C4H step impact the auxin response mechanism [52].

### Metabolite homeostasis may impact fruit breeding for quality

In the present work, we found that several most stable metabolites during tomato fruit development and between trusses correspond to metabolites crucial for fruit quality such as fructose, glucose, malate or GABA. The comparison of the CV tendencies observed for tomato with those for eight other fleshy fruit species ([53], Additional file 12) showed that the CVs between stages for malate were generally low during fruit development, and even below 25% in eggplant, apple and peach. Moreover, for pepper, eggplant, kiwi fruit, cucumber, apple, peach and clementine, malate CV between stages was lower than citrate CV between stages. Similarly, the CVs between stages for fructose and glucose were rather low (below or equal to 35%) in eggplant, cucumber and peach, and in eggplant, cucumber, apple and peach, respectively (Additional file 12). These features were independent of the climacteric or non-climacteric ripening mode of the fruit species.

Identifying such “housekeeping” metabolites provides knowledge about metabolism regulation but also potential information for fruit breeding for organoleptic or nutritional quality. Based on our results and the latter multispecies comparison, increasing fruit acidity seems more feasible by increasing citrate content (less homeostatic) than malate content (more homeostatic). For glucose and fructose, as their homeostasis and accumulation level during ripening depended on the species (Additional file 12), no general conclusion for fleshy fruits can be proposed. However, as for the majority of most stable metabolites almost no direct correlation between a metabolite and its specific pathway-related transcripts or proteins was observed in tomato, metabolomics-based biochemical phenotyping appears as a crucial complement to molecular breeding [54, 55].

## Conclusions

**Fig. 4:**
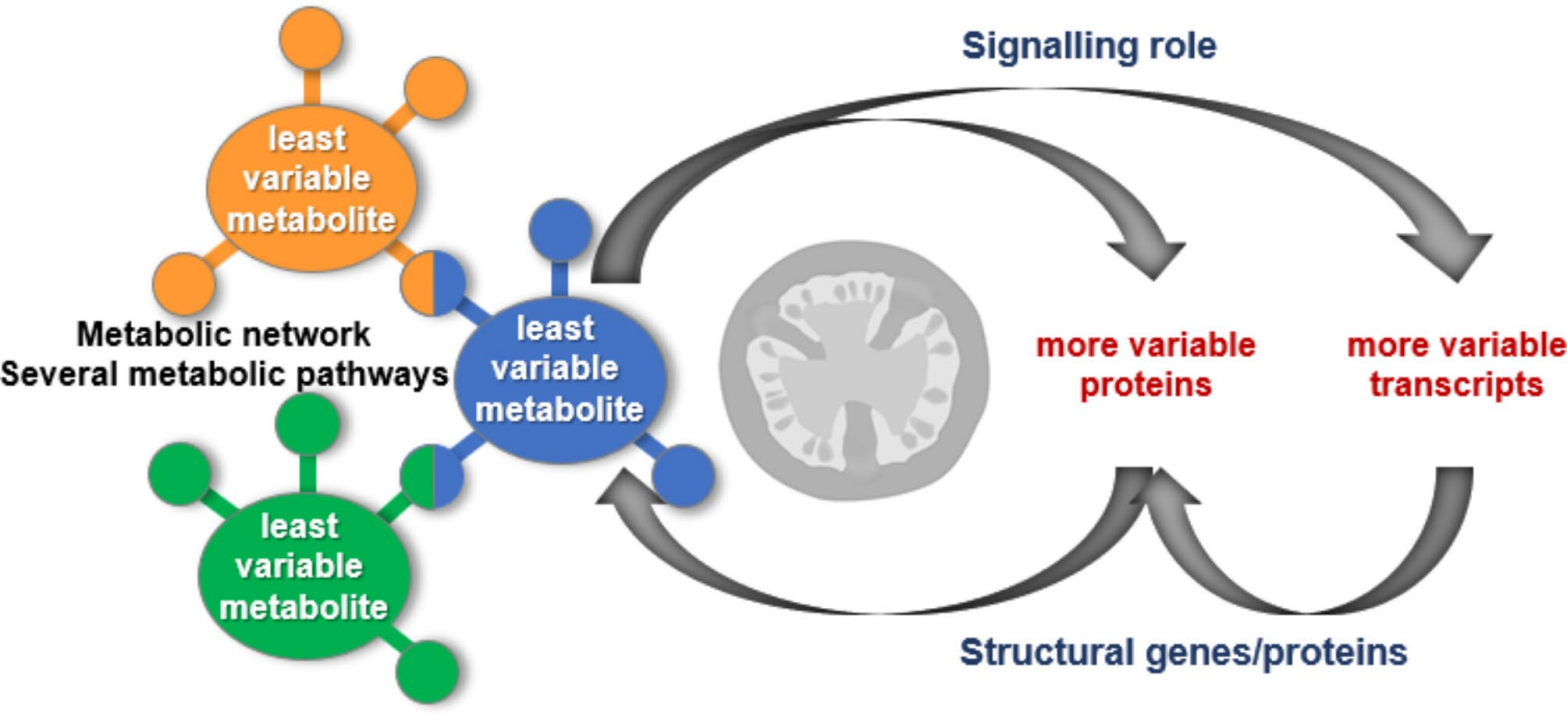
Schema of possible regulatory factors controlling least variable metabolites in tomato pericarp. We hypothesise that the least stable metabolites are hubs in metabolic networks and have a signalling role for the regulation of more variable proteins and transcripts in a complex regulatory network implicating several metabolic pathways

In this work, we tested a new way of analysing omics data by focusing on the most homeostatic metabolites. In agreement with our hypothesis that they might play a particular role during tomato fruit development, several of them proved to be hubs in metabolic pathways or had a signalling role. We investigated whether they were regulated either by stable proteins or transcripts, or by complex regulations involving coordinated changes in the contents of several proteins and transcripts (Fig. 4). The latter hypothesis was more in line with our data. To verify whether these trends are common to fleshy fruits, similar work should be performed on another fruit species.

## Materials and methods

### Plant material

Tomato plants (*Solanum lycopersicum* cv. Moneymaker, seeds provided by Dr Alisdair Fernie at Max Planck Institute of Molecular Plant Physiology) were grown under conditions of commercial production in a greenhouse. Fruits were harvested at nine stages of tomato fruit development (8, 15, 21, 28, 34, 42, 48, 50 and 53 DPA), on the 5th, 6th and 7th trusses. The fruits of the different trusses experienced slightly different microenvironmental and environmental conditions, including temperature that may modify their metabolism and composition. Fruit pericarp was taken, immediately frozen and analysed using metabolomic, proteomic and transcriptomics strategies. For details about samples, see [56].

### Compound and metabolome analyses

Metabolites, metabolite signatures and starch were determined in fresh-frozen or lyophilized samples using the following analytical strategies. Absolute concentrations were calculated for starch and for all identified metabolites whenever possible. The percent dry weight of each sample was determined using lyophilisation. All results are expressed on a fresh weight basis for all data analyses. However, to verify the possible impact of pericarp water content, the metabolome data were also expressed on a dry weight basis for its overview using a PCA analysis.

### Targeted analyses of starch

Starch contents were extracted from a previous study performed on the same samples [32] using determination in the pellets obtained after polar compound extraction with enzymatic analyses performed on a robotic Star/Starlet platform (Hamilton, Villebon sur Yvette, France) and spectrophotometers. After neutralization of the suspended pellet, starch was determined and expressed in glucose equivalents per g fresh weight (FW).

### Proton NMR profiling

NMR analyses of major polar compounds (major soluble sugars, organic acids, amino acids, amines) were performed on ethanolic extracts of 20-mg lyophilized samples, as previously described [2]. Absolute quantification of individual metabolites was achieved using a 500-MHz Avance III NMR spectrometer (Bruker Biospin, Wissembourg, France) equipped with a 5-mm ATMA BBI inverse probe and a BACS-120 autosampler using TopSpin IconNMR 3.0 software (Bruker Biospin, Karlsruhe, Germany) and the ERETIC II method (Digital ERETIC, Bruker TopSpin 3.0) for quantification. The analysis temperature was 300 K. A single pulse (zg) sequence was used for ^1^H-NMR acquisitions with the following parameters: 64 scans, a 6,002-Hz spectral width, 2.73-s acquisition time, 90° pulse angle (pulsecal), 25-s recycle delay, a fix receiver gain. The raw 1D ^1^H spectral data were processed with TopSpin 3.0 software. After Fourier transformation and a line broadening of 0.3 Hz on the obtained free induction decay (FID), the spectra were manually phased and calibrated at 0 ppm using the TSP signal. The baseline was manually corrected globally and locally using polynomial functions. Metabolite assignments were made by comparing proton chemical shifts with literature [2] or database values (in-house library, MERYB [57], HMDB [58], BMRB [59]), spiking and complementary 2D NMR experiments (heteronuclear single quantum coherence, HSQC; J-resolved spectroscopy, JRES; correlated spectroscopy, COSY) performed on representative samples. The areas of resonances of interest were integrated using the ‘Analytical Profiler’ mode of the AMIX software (version 3.9.14, Bruker). Metabolite quantification was performed using calibration curves, as previously described [2]. Unidentified metabolite resonances were named ‘Unk’ followed by the shape of the NMR pattern (S = singlet, D = doublet, M = multiplet) and by their chemical shift value.

### LC-MS-based targeted analyses

A first series of MS-based targeted analyses was performed on 20-mg methanolic extracts of fresh frozen powder using ion-pair reversed phase LC-MS/MS as described previously [60], to determine several organic acids, amino acids and intermediaries of central metabolism including sugar phosphates.

A second series of MS-based targeted analyses was performed on ethanolic extracts of fresh frozen powder using LC-HRMS/MS to determine several additional amino acids. Fifty mg FW were extracted with 300 µL ethanol/water (80:20 v/v). Ten µL of a mixture of ^13^C- and ^15^N-labelled internal standards (including arginine (^13^C_6_; ^15^N_4_), histidine (^13^C_6_; ^15^N_3_), lysine (^13^C_6_; ^15^N_2_), methionine (^13^C_5_; ^15^N), serine (^13^C_3_; ^15^N) from Cambridge Isotope Laboratories (Andover, USA) with purity between 98% and 99.9% for each compound) were added for quantification. Extraction was carried out at 80 °C for 20 min. Centrifugation was performed at 14,462 *g* for 5 min, and the supernatant was filtered at 0.2 μm and analysed by LC-HRMS/MS. For unlabelled citrulline and ornithine, standard solutions for calibration were prepared in acetonitrile/water (50:50 v/v) with concentrations ranging from 0.2 to 10 µg.mL^− 1^. All solutions were stored at -20 °C before use. Liquid chromatography was performed on a Dionex UHPLC Ultimate 3000 (Thermo Scientific, Villebon-sur-Yvette, France) equipped with a binary solvent delivery system, a sample manager, a column compartment and a diode array detector. Hydrophilic interaction liquid chromatography (HILIC) separation was performed on an Acclaim Mixed-Mode HILIC-1 column (2.1 × 150 mm; 3 μm, Dionex-Thermo Scientific, Courtaboeuf, France) equipped with an Acclaim Mixed-Mode HILIC-1 guard column (2.1 × 10 mm; 5 μm, Dionex). Solvent A was composed of 20 mM of ammonium formate in water at pH 4 (95%) and 5% acetonitrile, and solvent B was acetonitrile. The gradient started at 95% B for 2.5 min, followed by a linear gradient down to 75% B for 3 min, and a second linear gradient for 2.5 min to 0% B. The mobile phase remained at 0% B for 5 min and then returned to the initial conditions in 0.5 min. The column was equilibrated for 7.5 min in the initial conditions (95% B) prior to the next injection, for a total run time of 21 min. Flow rate was 0.35 mL.min^− 1^ and column temperature was maintained at 30 °C. The autosampler temperature was maintained at 4 °C and the injection volume was 5 µL. The UHPLC system was coupled with an LTQ-Orbitrap Elite mass spectrometer (Thermo Scientific, Bremen, Germany). A HESI II interface was used and analyses were performed in both positive and negative modes. Acquisition was performed in full scan mode with a resolving power of 120,000 FWHM at *m/z* 400 in the scan range of *m/z* 50-1000. ESI parameters were as follows: heater temperature 350 °C, capillary temperature 350 °C, sheath gas 45 (arbitrary units), auxiliary gas 15 (arbitrary units), S-Lens 60 V, spray voltage: 3.2 kV in ESI^+^ and 2.5 kV in ESI^−^. Data were recorded using Xcalibur software (Thermo Scientific, Bremen, Germany) and QuanBrowser software was used for quantification. Chromatograms of targeted compounds were extracted using exact *m/z* of the protonated or deprotonated molecule with a 10-ppm mass window tolerance. Acetonitrile, formic acid and ammonium formate were of LC-MS grade and purchased from Sigma-Aldrich® (Steinheim, Germany). Ethanol (absolute, ≥ 99.8%) was also obtained from Sigma-Aldrich® (Steinheim, Germany).

### LC-DAD targeted analyses of isoprenoids

Isoprenoids were determined on the chloroform phase of extracts of fresh frozen samples, dried under a stream of nitrogen and resuspended in ethyl acetate using LC-DAD, as previously described [2].

### LC-MS-based untargeted analyses

MS-based untargeted analyses were performed on methanolic extracts of lyophilized powder to obtain signatures of specialized metabolites, such as phenolics and glycoalkaloids. Extraction was performed on 20 mg dry weight (DW) using a MeOH/H_2_O (70/30 v/v) solvent containing 0.1% formic acid and 1.37 mM of methyl vanillate (used to verify injection) in an ice-cold ultrasonic bath followed by centrifugation at 14,462 *g* for 5 min. Supernatants were filtered (0.22 μm, PVDF, Millipore, Cork, Ireland) and transferred into HPLC vials. The preparation of an extraction blank was also performed to eliminate contaminants during data processing. A quality control (QC) sample was made up from the mixture of 50 µL of each sample extract. Five µL of each extract were injected. The analytes were separated by HPLC (Thermoscientific Ultimate 3000, Dionex, CA, USA) on a C18 reverse phase column (Gemini, 150 mm x 2.1 mm, 3 μm, Phenomenex, CA, USA). The gradient used was as previously described [61], with a flow rate of 350 µL/min. The compounds were ionized by an electrospray source in positive (4.5 kV) mode and detected by a hybrid QTOF mass analyser, micrOTOF-Q (Bruker Daltonics, Bremen, Germany). The mass-to-charge ratio ion scan was *m/z* 50 to *m/z* 1,500 with an acquisition frequency of 2 Hz and a resolving power of 15,000 at *m/z* 922. The nebulizer gas was at a pressure of 2.4 bar and dry gas flow was 8 L/min at a temperature of 190 °C. The samples were maintained at a temperature of 6 °C in the autosampler. The QC were injected every 10 samples to check for measurement stability.

After acquisition, the data set was converted into mzML format and exported to the W4M platform [62] to process the data using XCMS for variable filtration, identification of peaks, clustering of peaks, correction of retention times and generation of a data matrix. This matrix was normalized with the weights used for the extraction of each sample. The variables present in the blanks were eliminated, followed by those with a coefficient of variation between the QCs greater than 50%. The final matrix for data mining had 1,166 metabolite signature variables. Thirteen of them were annotated based on published data [61] and public spectral databases (Massbank [63, 64], mzCloud [65]). Further annotations of selected variables were attempted after data statistical analysis, based on SmartFormula algorithm (Bruker Daltonics, Bremen, Germany) and PubChem database [66] search.

### Proteome and transcriptome data

The proteome and transcriptome data, issued from the same fruit samples as those used for metabolomics, were from the deposited data mentioned in the study of [56]. For LC-MS/MS-based proteomics, peptide ions were quantified using extracted ion chromatograms. The peptide intensities of each sample were normalized based on the intensities of a reference sample. Proteins were quantified. Absolute quantification was approximated based on the ‘Total Protein Amount’ approach [67]. To determine the absolute concentration of transcripts after transcriptome sequencing, internal standards (AM 1780, Ambion by Life Technologies, Array Control RNA spikes, Invitrogen™) at selected concentrations were spiked in the plant extracts at the beginning of the RNA purification process. Therefore, both proteome and transcriptome data are absolute quantification data expressed as µmol per g FW. Protein and transcript sequences were recovered from the Sol Genomics Network database ([68] ITAG2.4 gene models) and annotated with Mercator4 v4.0 [69, 70].

### Statistical analyses

A PCA was performed on the metabolome data set after mean-centring and unit-variance scaling with BioStatFlow based on R scripts (v2.9, [71, 72]). The most stable variables in the metabolome dataset were selected based on their CV between trusses and that between stages. The CV between trusses was the mean of all CVs between trusses calculated per stage. The CV between stages was calculated from the means of the three trusses per stage of development. The thresholds used for variable selection were CV of stage means below 25%, and the mean of the nine CVs between trusses per stage below 25%. After elimination of redundancy in the MS-based signatures present in this selected set (manual filtration of fragments, adducts and isotopes), this set constituted the most stable variables of interest.

First, we examined the most stable proteome and transcriptome variables based on their CVs between trusses and between stages, to verify any putative functional link with the most stable metabolites. For proteome and transcriptome, the CVs between trusses and between stages per functional category were calculated as the mean of the CVs of the proteins or transcripts belonging to this functional category.

Second, in line with possible metabolic regulations, the set of most stable metabolite variables was combined with the entire proteome or transcriptome data with the mixOmics package of R [73], using the DIABLO application [74] for an sPLS analysis with LASSO penalization on the loading vectors of the latent variables. We used the regression mode to explore the links between the most stable metabolome dataset and the proteome or transcriptome one, and the sparse mode to perform variable selection in the proteome or transcriptome datasets. We did not use this approach to predict a dataset or ‘block’ from another data block, but rather to select variables that covary between the most stable variables and one of the other omic datasets. The annotations of the selected proteins and genes from Mercator4 were used to calculate category enrichments using PageMan ([75], Fisher’s tests with FDR correction).

To study the links between each of the most stable metabolite variables and all the variables of the other two omics datasets in a metabolite by metabolite approach, we calculated Spearman correlation coefficients and their corresponding p-values with R scripts. We selected the correlation pairs with a stringent *P* < 0.0001 threshold without correcting for multiple testing, as the correction effect would differ between the proteome (2,282 variables) and transcriptome (23,631 variables) datasets. The annotation of the selected proteins and genes from Mercator4 were used to calculate category enrichments for both positive and negative correlations using PageMan [75]. We also used an approach focusing on the known pathways involving each most stable metabolite. We looked for each metabolite’s pathways in the tomato SolCyc biochemical pathways database [76, 77] and found the included gene/protein accession numbers. This list was then refined using the BIN classification from the Mercator4 annotation [69] of our dataset to remove gene/protein with uncertain annotations and add missing ones. Spearman correlation coefficients were calculated from the most stable metabolites and the transcripts and proteins of the corresponding pathways.

## Electronic supplementary material

Below is the link to the electronic supplementary material.

Supplementary Material 1

Supplementary Material 2

## Data Availability

The metabolome data and their metadata have been deposited in https://entrepot.recherche.data.gouv.fr open repository with a doi (https://doi.org/10.57745/7OXXLB).

## Abbreviations

CV: coefficient of variation
DAD: diode array detection
DHAP: dihydroxyacetone-phosphate
DPA: days post-anthesis
DW: dry weight
FDR: false discovery rate
FW: fresh weight
GABA: γ-aminobutyric acid
HILIC: hydrophilic interaction liquid chromatography
LC-MS/MS: liquid chromatography coupled to tandem mass spectrometry
MSI: Metabolomics Standards Initiative
PC: principal component
PCA: principal component analysis
QC: quality control
QTOF: quadrupole time-of flight
sPLS: sparse partial-least-square analysis
