# Supplementary material for: Multi-omics quantitative data of tomato fruit unveils regulation modes of least variable metabolites
Source: BMC Plant Biol. 2023 Jul 22;23:365. doi: 10.1186/s12870-023-04370-0 (PMC10362748; doi:10.1186/s12870-023-04370-0)
Supplement: Supplementary file 1 — Supplementary Material 1 [file 12870_2023_4370_MOESM1_ESM.pptx]

## Slide 1
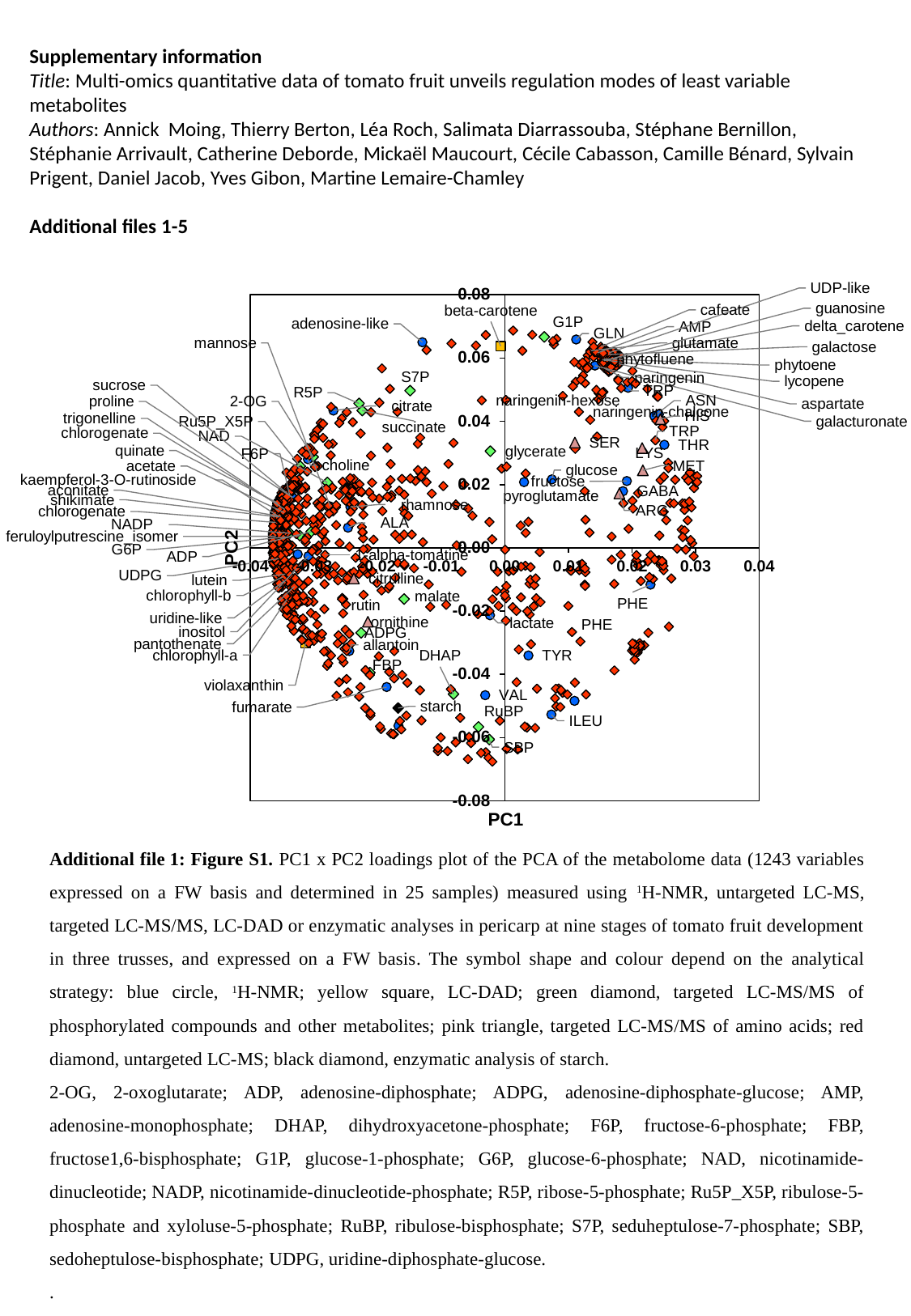

Supplementary information
Title: Multi-omics quantitative data of tomato fruit unveils regulation modes of least variable metabolites
Authors: Annick Moing, Thierry Berton, Léa Roch, Salimata Diarrassouba, Stéphane Bernillon, Stéphanie Arrivault, Catherine Deborde, Mickaël Maucourt, Cécile Cabasson, Camille Bénard, Sylvain Prigent, Daniel Jacob, Yves Gibon, Martine Lemaire-Chamley
Additional files 1-5
Additional file 1: Figure S1. PC1 x PC2 loadings plot of the PCA of the metabolome data (1243 variables expressed on a FW basis and determined in 25 samples) measured using 1H-NMR, untargeted LC-MS, targeted LC-MS/MS, LC-DAD or enzymatic analyses in pericarp at nine stages of tomato fruit development in three trusses, and expressed on a FW basis. The symbol shape and colour depend on the analytical strategy: blue circle, 1H-NMR; yellow square, LC-DAD; green diamond, targeted LC-MS/MS of phosphorylated compounds and other metabolites; pink triangle, targeted LC-MS/MS of amino acids; red diamond, untargeted LC-MS; black diamond, enzymatic analysis of starch.
2-OG, 2-oxoglutarate; ADP, adenosine-diphosphate; ADPG, adenosine-diphosphate-glucose; AMP, adenosine-monophosphate; DHAP, dihydroxyacetone-phosphate; F6P, fructose-6-phosphate; FBP, fructose1,6-bisphosphate; G1P, glucose-1-phosphate; G6P, glucose-6-phosphate; NAD, nicotinamide-dinucleotide; NADP, nicotinamide-dinucleotide-phosphate; R5P, ribose-5-phosphate; Ru5P_X5P, ribulose-5-phosphate and xyloluse-5-phosphate; RuBP, ribulose-bisphosphate; S7P, seduheptulose-7-phosphate; SBP, sedoheptulose-bisphosphate; UDPG, uridine-diphosphate-glucose.
.

## Slide 2
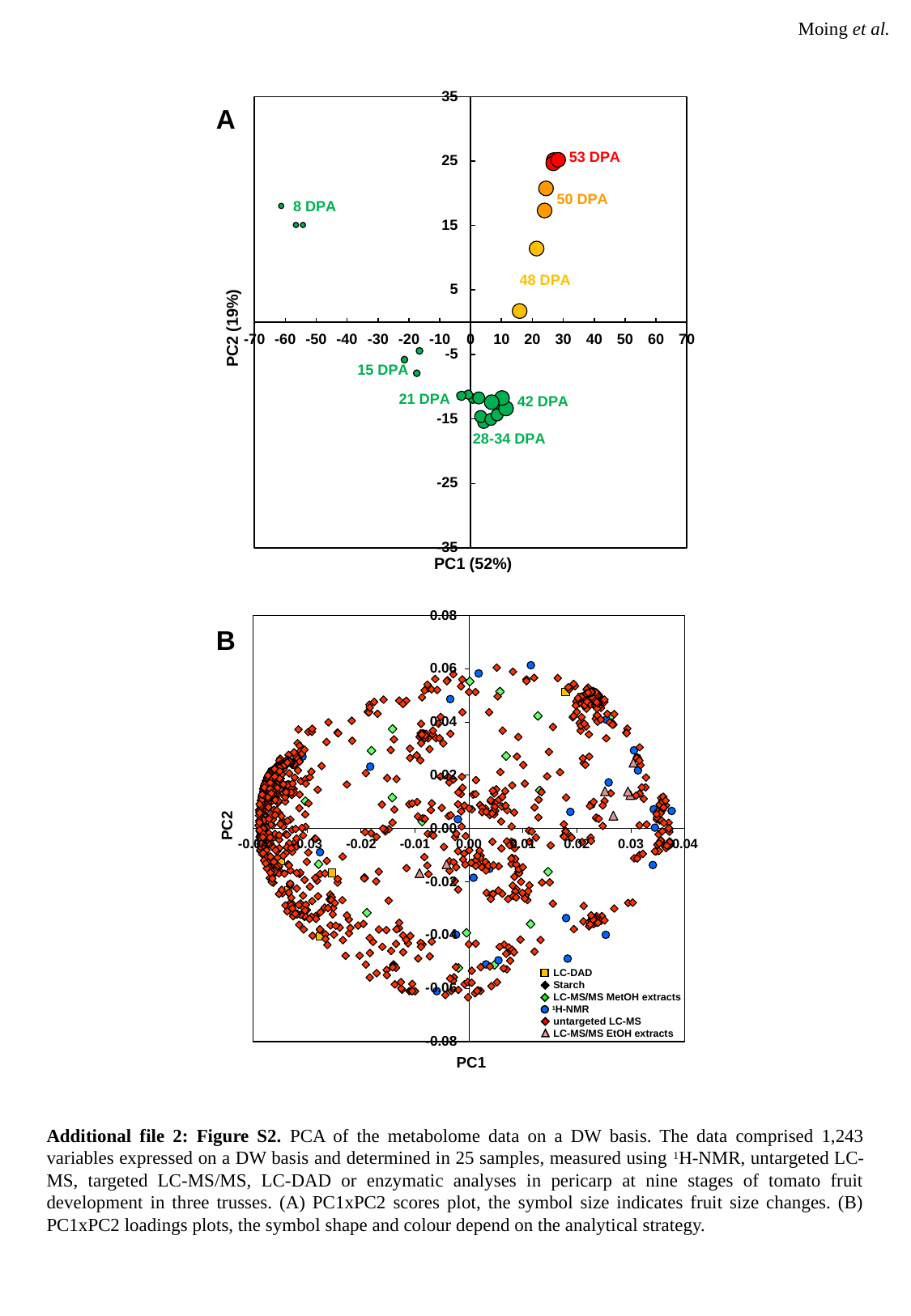

Moing et al.
A
B
 LC-DAD
 Starch
 LC-MS/MS MetOH extracts
 1H-NMR
 untargeted LC-MS
 LC-MS/MS EtOH extracts
Additional file 2: Figure S2. PCA of the metabolome data on a DW basis. The data comprised 1,243 variables expressed on a DW basis and determined in 25 samples, measured using 1H-NMR, untargeted LC-MS, targeted LC-MS/MS, LC-DAD or enzymatic analyses in pericarp at nine stages of tomato fruit development in three trusses. (A) PC1xPC2 scores plot, the symbol size indicates fruit size changes. (B) PC1xPC2 loadings plots, the symbol shape and colour depend on the analytical strategy.

## Slide 3
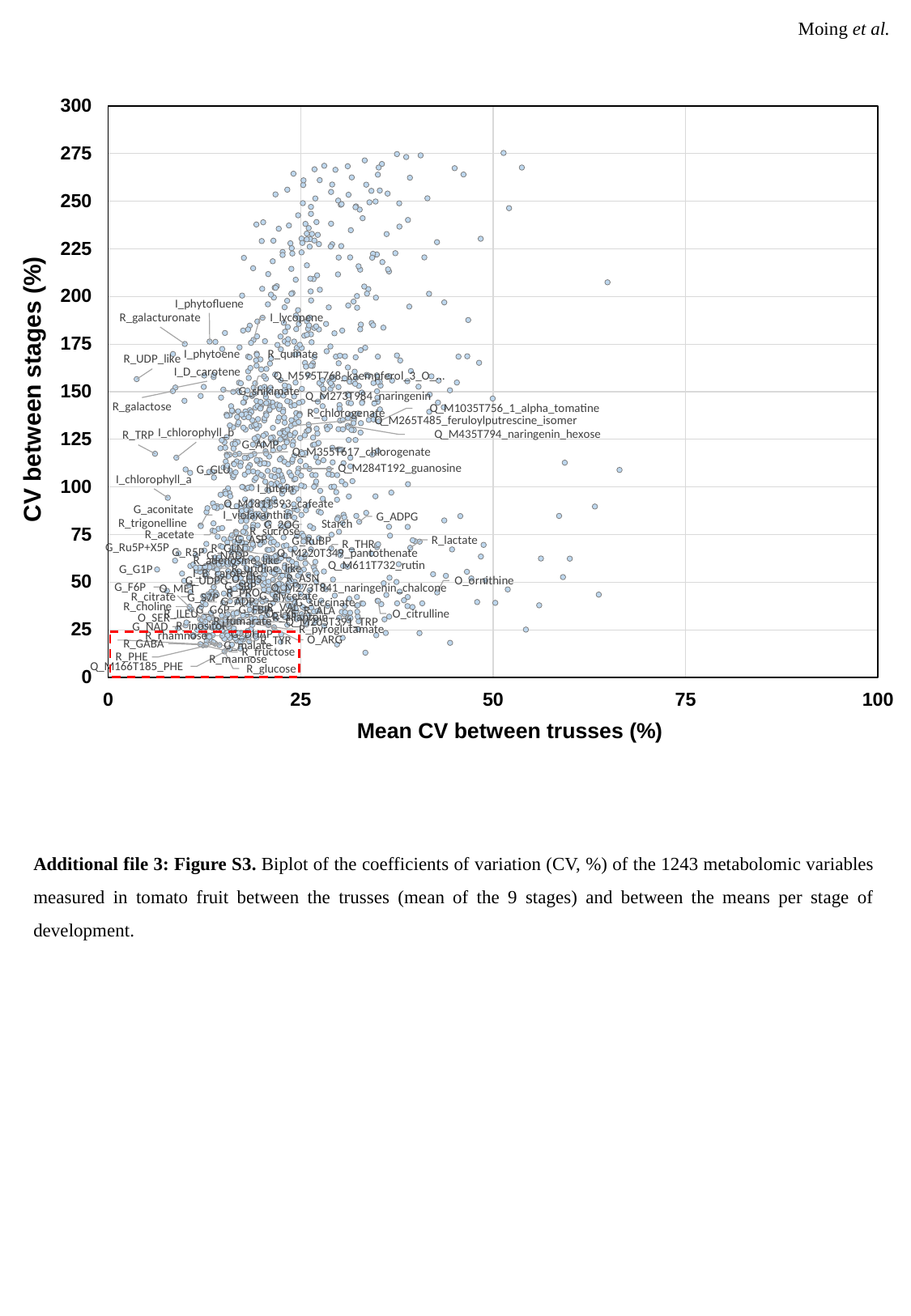

Moing et al.
Additional file 3: Figure S3. Biplot of the coefficients of variation (CV, %) of the 1243 metabolomic variables measured in tomato fruit between the trusses (mean of the 9 stages) and between the means per stage of development.

## Slide 4
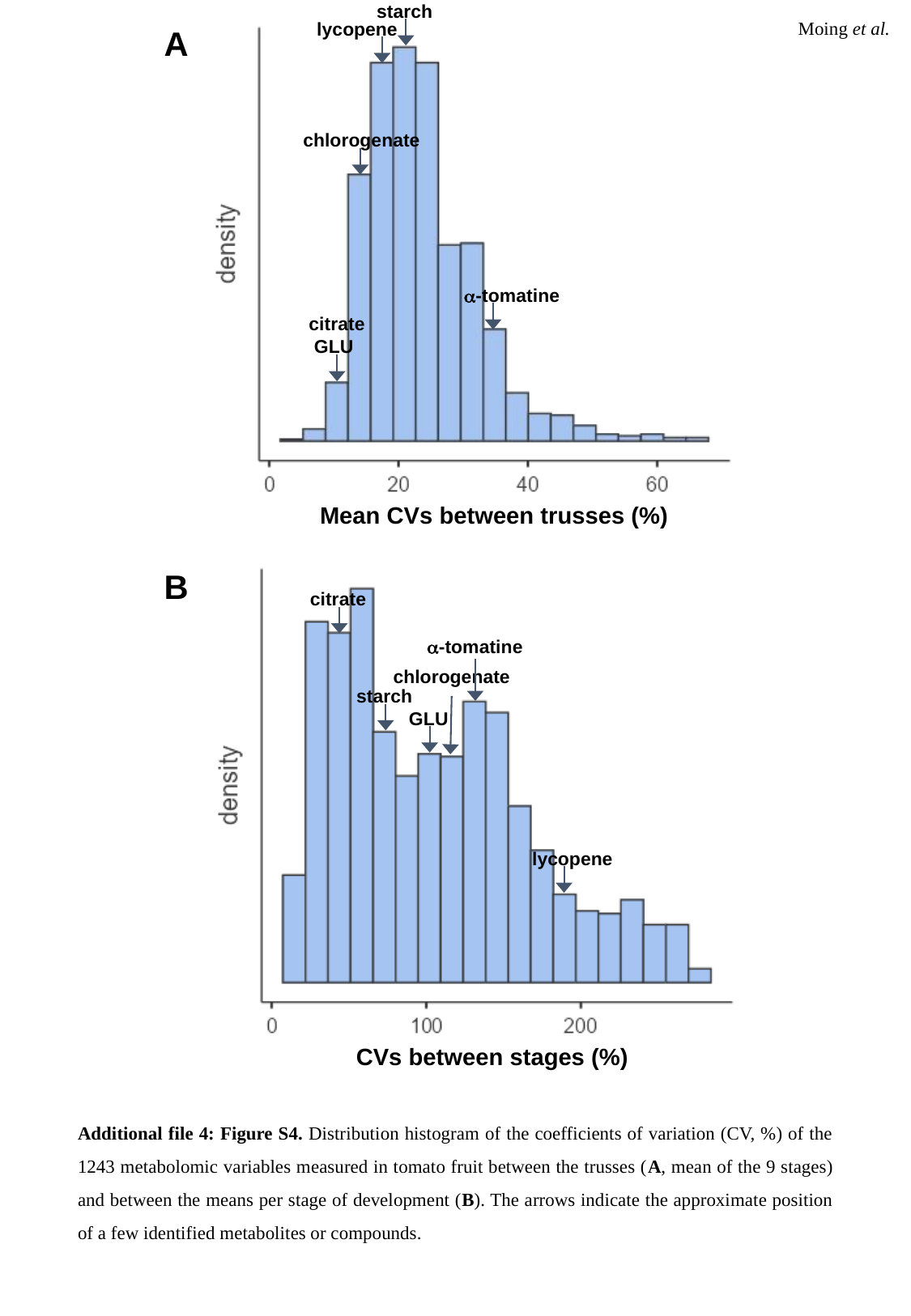

starch
Moing et al.
lycopene
A
chlorogenate
a-tomatine
citrate
 GLU
Mean CVs between trusses (%)
B
citrate
a-tomatine
chlorogenate
starch
GLU
lycopene
CVs between stages (%)
Additional file 4: Figure S4. Distribution histogram of the coefficients of variation (CV, %) of the 1243 metabolomic variables measured in tomato fruit between the trusses (A, mean of the 9 stages) and between the means per stage of development (B). The arrows indicate the approximate position of a few identified metabolites or compounds.

## Slide 5
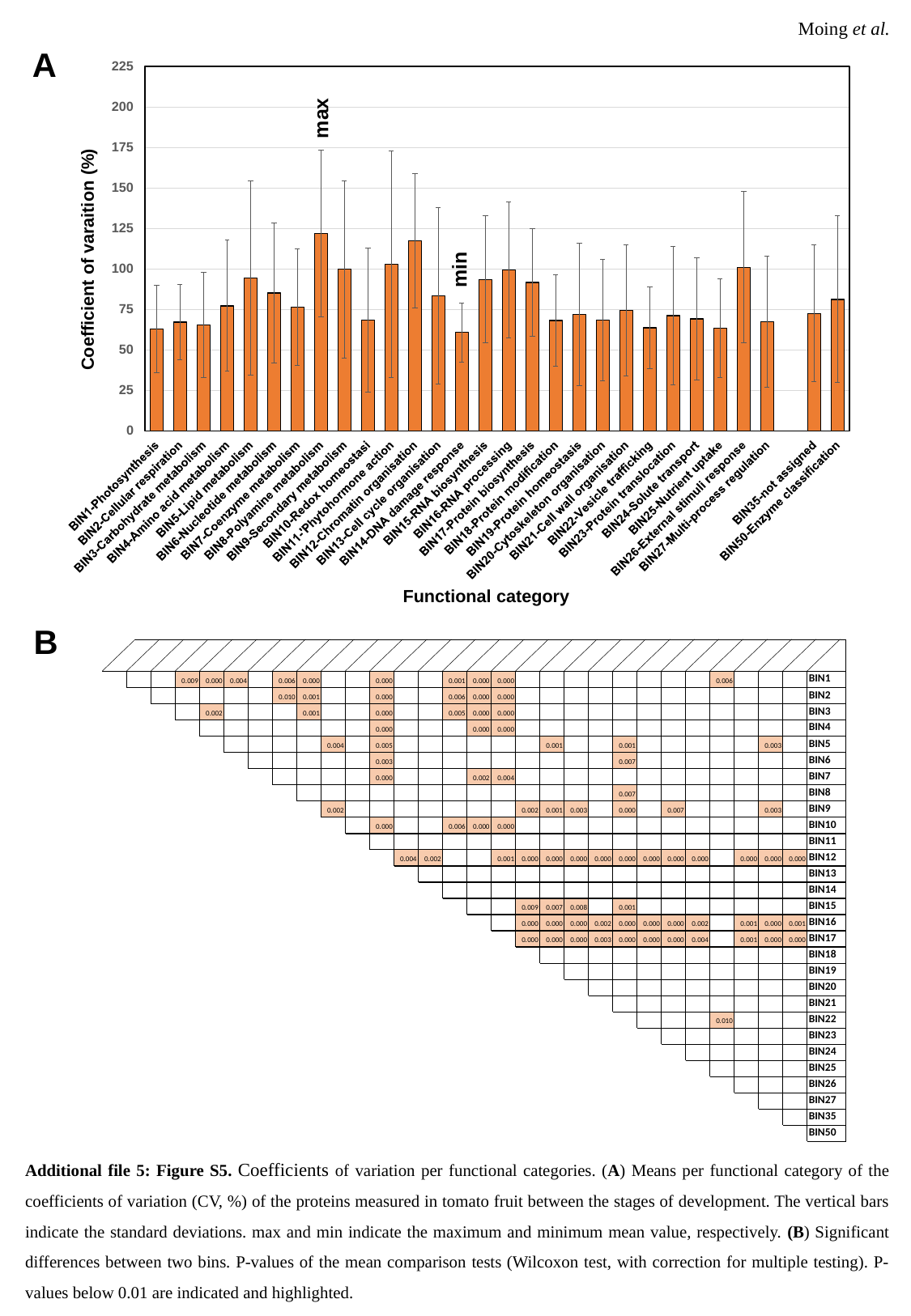

Moing et al.
A
max
min
B
Additional file 5: Figure S5. Coefficients of variation per functional categories. (A) Means per functional category of the coefficients of variation (CV, %) of the proteins measured in tomato fruit between the stages of development. The vertical bars indicate the standard deviations. max and min indicate the maximum and minimum mean value, respectively. (B) Significant differences between two bins. P-values of the mean comparison tests (Wilcoxon test, with correction for multiple testing). P-values below 0.01 are indicated and highlighted.

## Slide 6
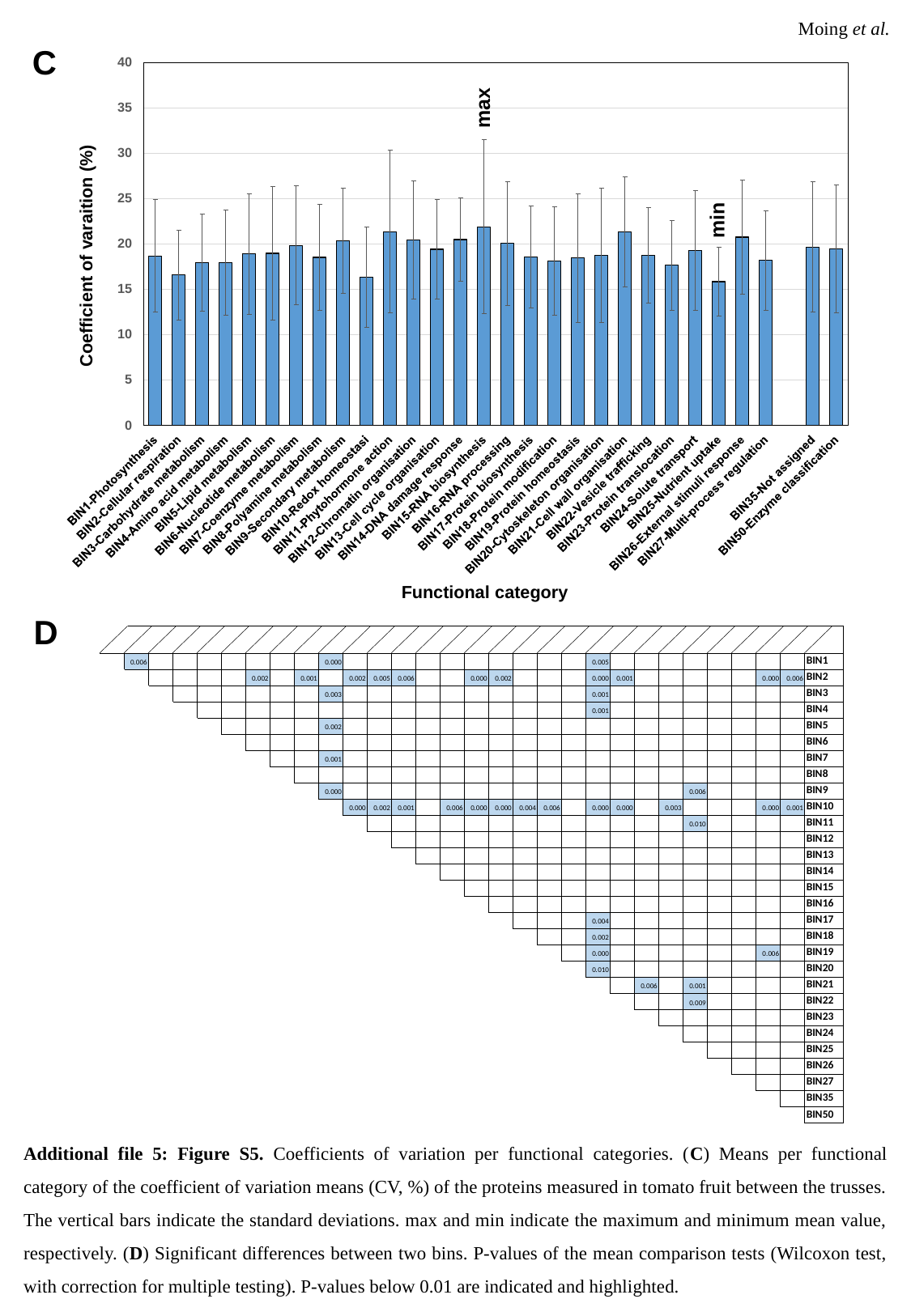

Moing et al.
C
max
min
D
Additional file 5: Figure S5. Coefficients of variation per functional categories. (C) Means per functional category of the coefficient of variation means (CV, %) of the proteins measured in tomato fruit between the trusses. The vertical bars indicate the standard deviations. max and min indicate the maximum and minimum mean value, respectively. (D) Significant differences between two bins. P-values of the mean comparison tests (Wilcoxon test, with correction for multiple testing). P-values below 0.01 are indicated and highlighted.

## Slide 7
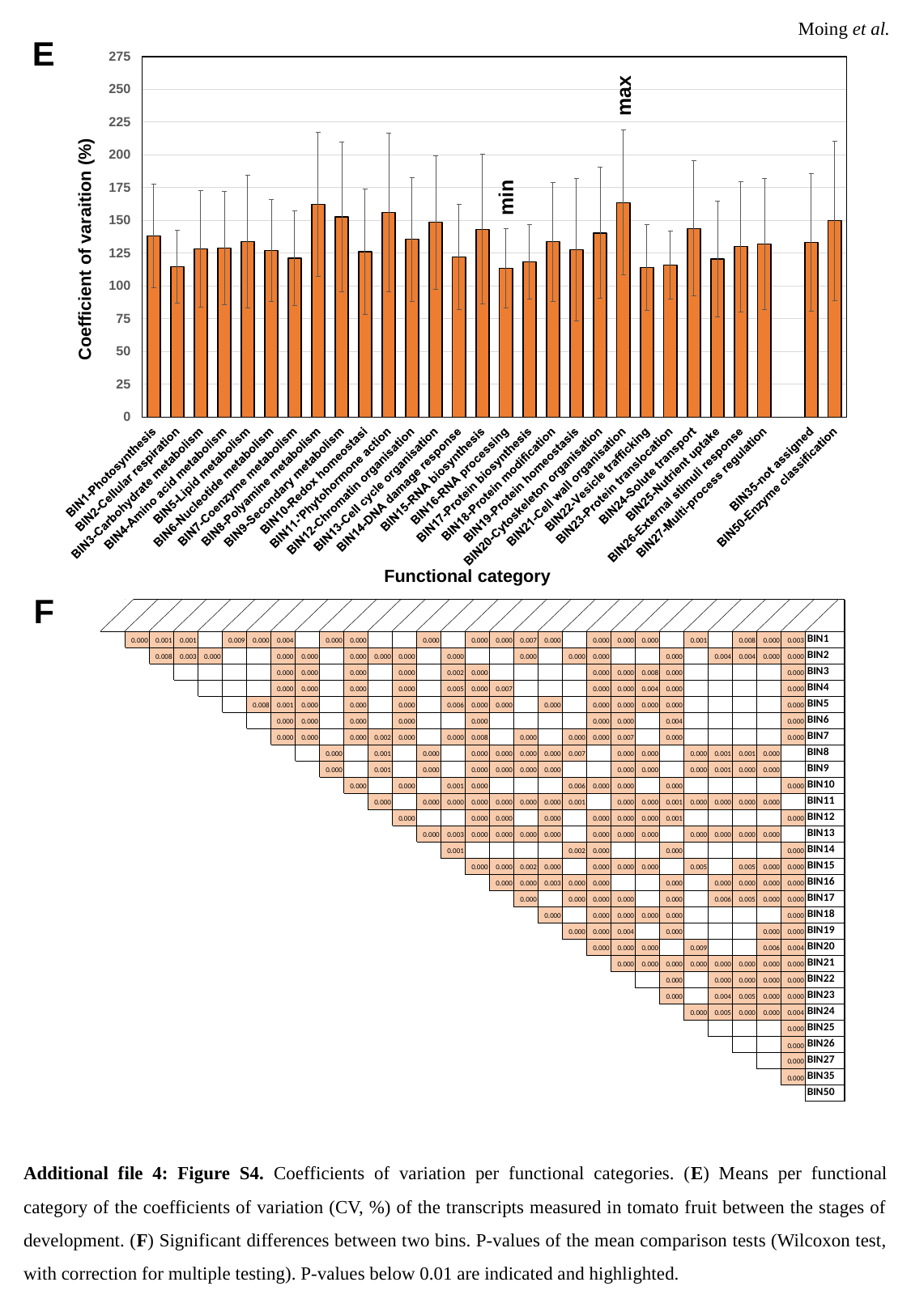

Moing et al.
E
max
min
F
Additional file 4: Figure S4. Coefficients of variation per functional categories. (E) Means per functional category of the coefficients of variation (CV, %) of the transcripts measured in tomato fruit between the stages of development. (F) Significant differences between two bins. P-values of the mean comparison tests (Wilcoxon test, with correction for multiple testing). P-values below 0.01 are indicated and highlighted.

## Slide 8
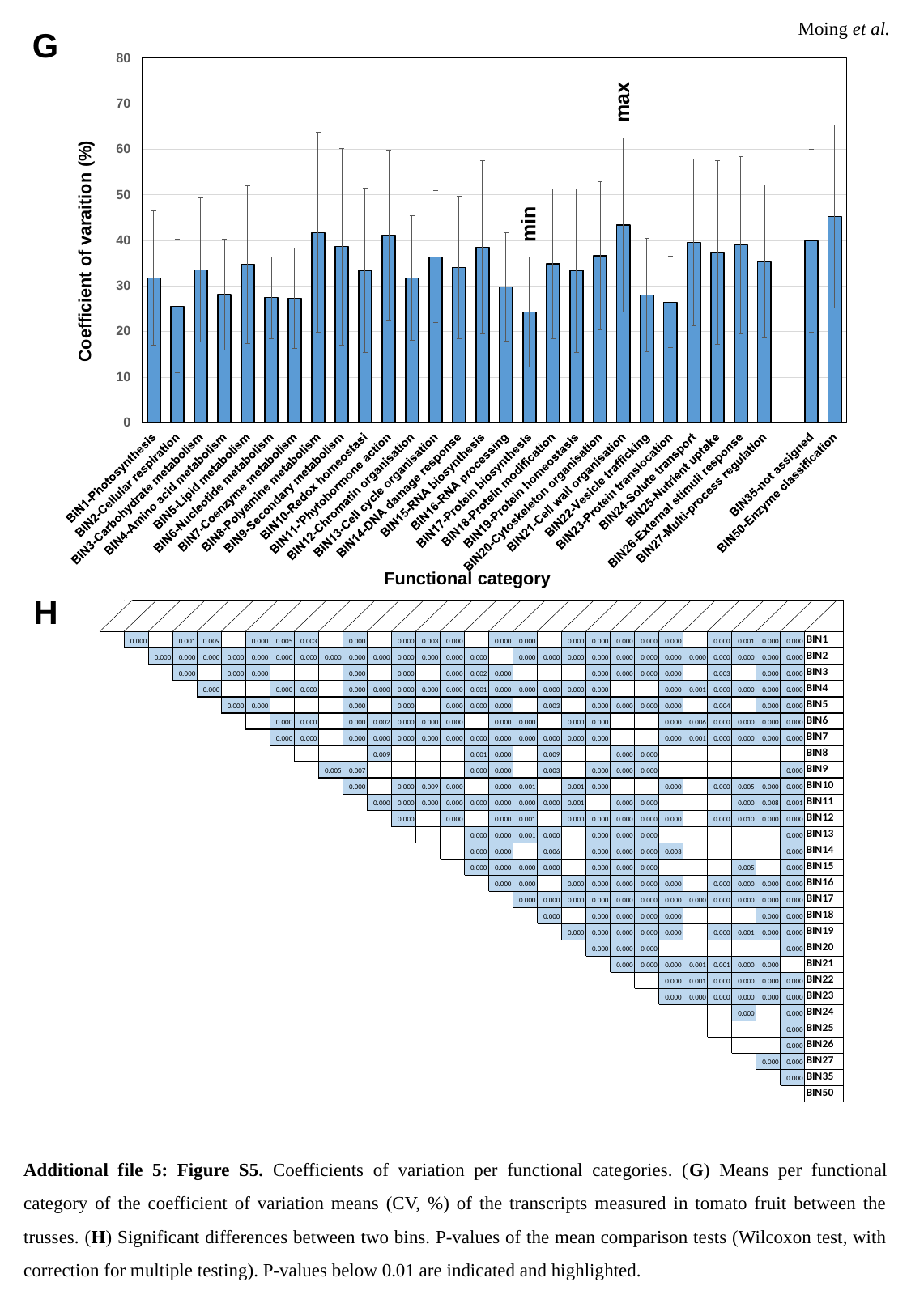

Moing et al.
G
max
min
H
Additional file 5: Figure S5. Coefficients of variation per functional categories. (G) Means per functional category of the coefficient of variation means (CV, %) of the transcripts measured in tomato fruit between the trusses. (H) Significant differences between two bins. P-values of the mean comparison tests (Wilcoxon test, with correction for multiple testing). P-values below 0.01 are indicated and highlighted.

## Slide 9
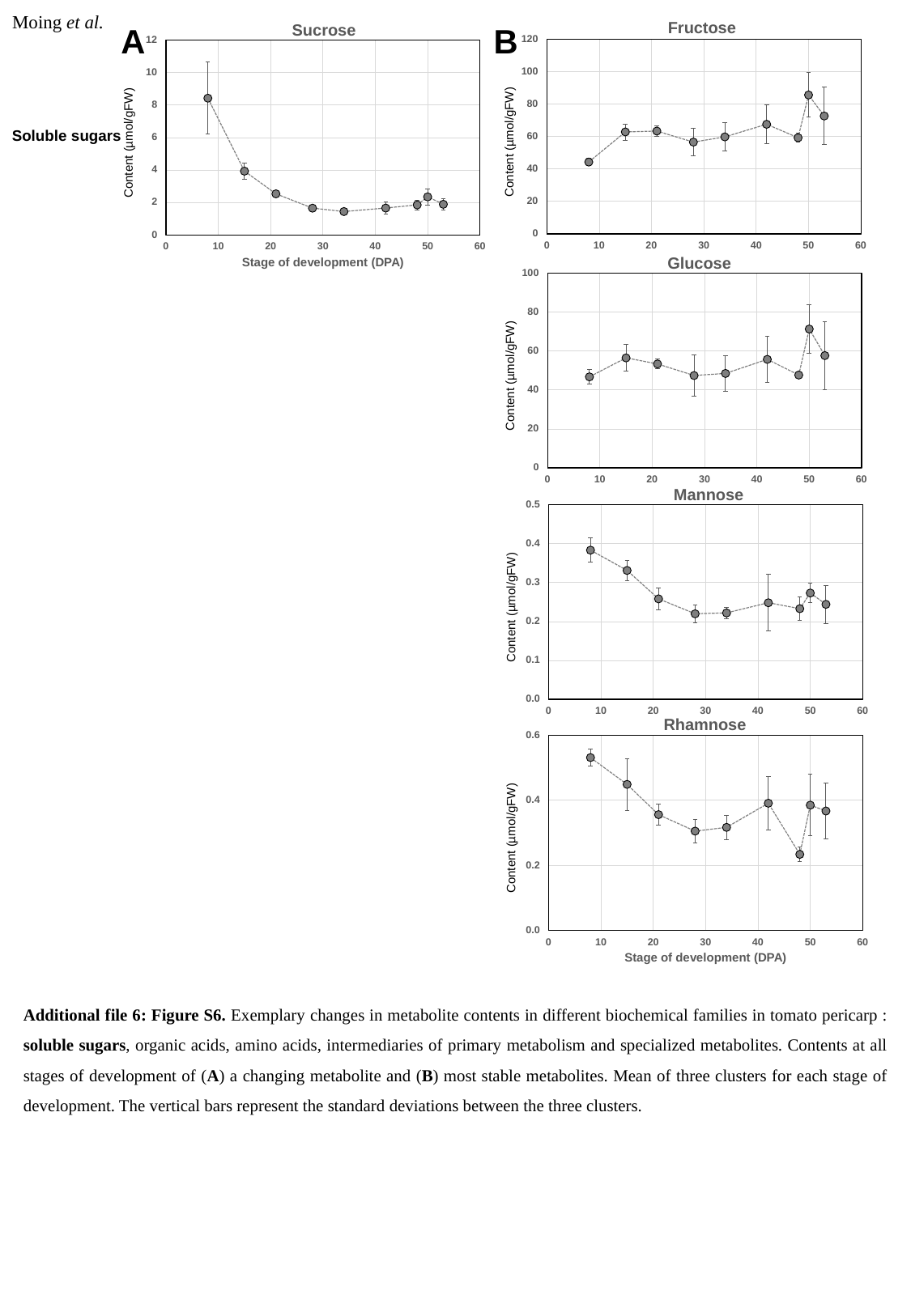

Moing et al.
A
B
Soluble sugars
Additional file 6: Figure S6. Exemplary changes in metabolite contents in different biochemical families in tomato pericarp : soluble sugars, organic acids, amino acids, intermediaries of primary metabolism and specialized metabolites. Contents at all stages of development of (A) a changing metabolite and (B) most stable metabolites. Mean of three clusters for each stage of development. The vertical bars represent the standard deviations between the three clusters.

## Slide 10
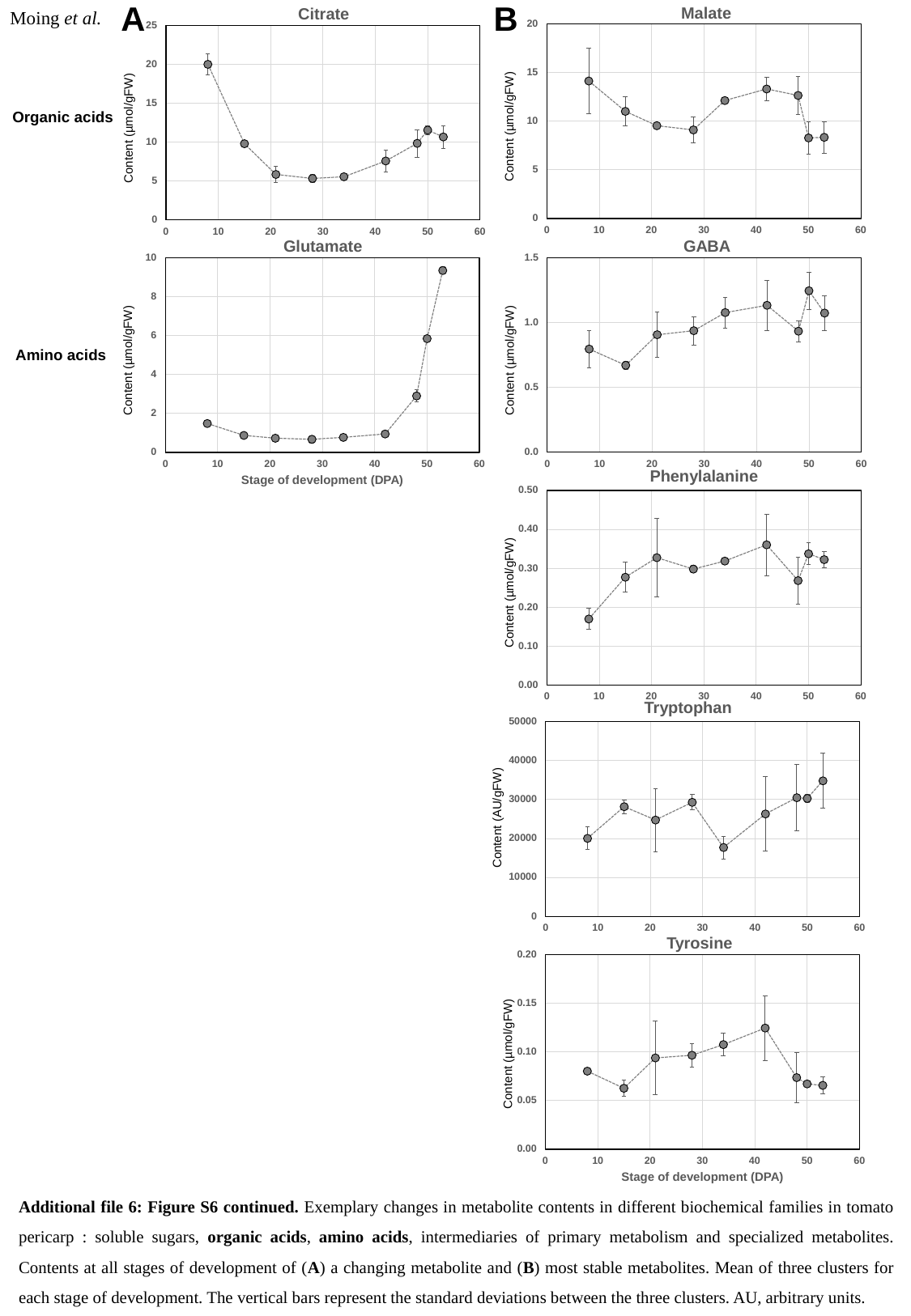

A
B
Moing et al.
Organic acids
Amino acids
Additional file 6: Figure S6 continued. Exemplary changes in metabolite contents in different biochemical families in tomato pericarp : soluble sugars, organic acids, amino acids, intermediaries of primary metabolism and specialized metabolites. Contents at all stages of development of (A) a changing metabolite and (B) most stable metabolites. Mean of three clusters for each stage of development. The vertical bars represent the standard deviations between the three clusters. AU, arbitrary units.

## Slide 11
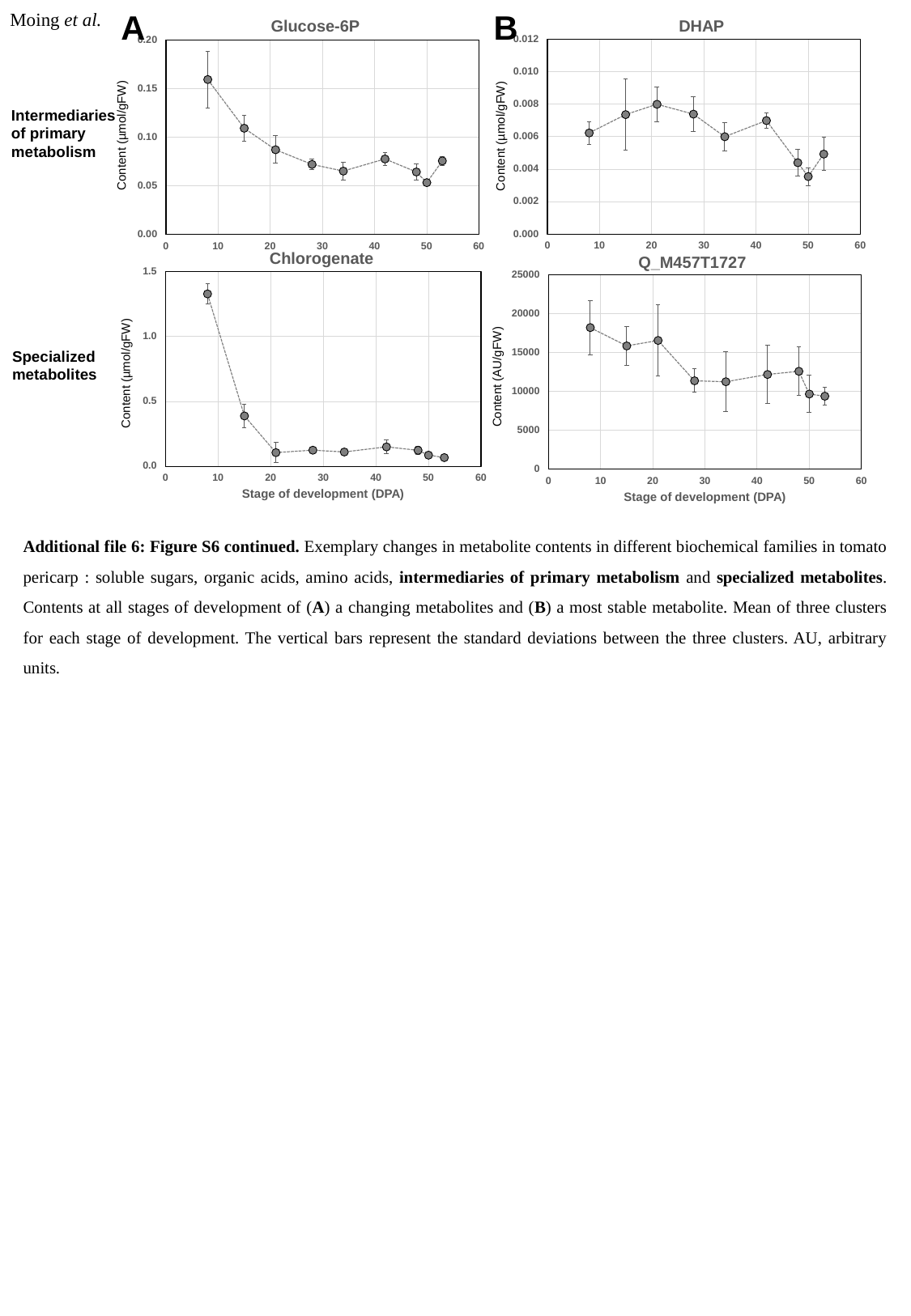

A
B
Moing et al.
Intermediaries
of primary
metabolism
Specialized
metabolites
Additional file 6: Figure S6 continued. Exemplary changes in metabolite contents in different biochemical families in tomato pericarp : soluble sugars, organic acids, amino acids, intermediaries of primary metabolism and specialized metabolites. Contents at all stages of development of (A) a changing metabolites and (B) a most stable metabolite. Mean of three clusters for each stage of development. The vertical bars represent the standard deviations between the three clusters. AU, arbitrary units.
